# Distinct virulent network between healthcare- and community-associated *Staphylococcus aureus* based on proteomic analysis

**DOI:** 10.1186/s12014-017-9178-5

**Published:** 2018-01-08

**Authors:** Lei He, Hongwei Meng, Qian Liu, Mo Hu, Yanan Wang, Xiaoying Chen, Xiaoyun Liu, Min Li

**Affiliations:** 1 Department of Laboratory Medicine, Ren Ji Hospital, School of Medicine, Shanghai Jiao Tong University, Shanghai, 200127 China; 2 Institute of Analytical Chemistry and Synthetic and Functional Biomolecules Center, College of Chemistry and Molecular Engineering, Peking University, Beijing, China

**Keywords:** *Staphylococcus aureus*, Virulent mechanism, Healthcare-associated, Community-associated, Proteomic analysis

## Abstract

**Background:**

*Staphylococcus aureus* (*S. aureus* or SA) is a leading cause of healthcare-associated (HA-) and community-associated (CA) infection. HA-SA isolates usually cause nosocomial pneumonia, bloodstream infections, catheter-related urinary tract infections, *etc*. On the other hand, CA-SA isolates usually cause highly fatal diseases, such as SSTIs as well as post influenza necrotic hemorrhagic pneumonia. The differences of the infection types are partially due to the unique characteristics between HA-SA and CA-SA isolates. For example, HA-SA isolates showed strong adherence to host epithelial cells, while CA-SA isolates displayed higher virulence due to the increased activity of the important quorum-sensing system accessory gene regulator (*agr*). Thus, the aim of this study was to characterize the proteomic difference between HA-SA and CA-SA lineage.

**Methods:**

In this study, the extracted peptides from those representative strains were analyzed by LC-MS/MS. The protein-protein interaction network was constructed by bioinformatics and their expressions were verified by RT-PCR and Western blot.

**Results:**

We demonstrated that Agr system (AgrA and AgrC) and its interactive factors (PhoP, SrrB, YycG, SarX, SigB and ClpP) based on the protein–protein interaction network were expressed significantly higher in the epidemic Chinese CA-SA lineage ST398 compared to HA-SA lineage ST239 by LC-MS/MS. We further verified the increased transcription of all these genes in ST398 by RT-PCR, suggesting that the higher expression of these genes/proteins probably play role in the acute infection of CA-SA. Moreover, surface-related proteins (FnbpA, SpA, Atl, ClfA, IsaA, IsaB, LtaS, SsaA and Cna) that are repressed by the Agr system have significantly higher expression in the epidemic Chinese HA-SA clone ST239 in comparison to CA-SA lineage ST398 by LC-MS/MS. Furthermore, we confirmed the significantly increased expression of two important adhesive proteins (Atl and ClfA) in ST239 by Western blot, which may contribute to the durative infection of HA-SA.

**Conclusion:**

The results suggest that the different proteomic profile, at least partially, contribute to the pathogenic differences between HA-SA and CA-SA.

**Electronic supplementary material:**

The online version of this article (10.1186/s12014-017-9178-5) contains supplementary material, which is available to authorized users.

## Background

*Staphylococcus aureus* has been a major cause of human infections for a long time [1]. In general, healthcare-associated (HA)-SA infections primarily occur among inpatients [2, 3] and typically result in septicemia, pneumonia and device-related infections. Due to the greater resistance rates for majority of antibiotics, healthcare-associated SA (HA)-SA infections are likely to promote the emergence of methicillin-resistant SA (MRSA), therefore causing increased hospital duration and higher hospital charges than methicillin-sensitive SA (MSSA) infections [4, 5]. By contrast, community-associated (CA)-SA infections caused by both MRSA and MSSA isolates mostly occur in healthy individuals and cause severe skin and soft-tissue infections (SSTIs) and, less frequently, necrotizing pneumonia in the community [3, 6].

The MRSA challenge in China is also a serious health issue both in healthcare and community environments. Recent epidemiological surveys have demonstrated that ST239-SCC*mec*III is the dominant isolate of HA-MRSA in China [7, 8], with ST5-SCC*mec*II ranked second [9, 10]. Comparatively, data on CA-SA in China are still limited. Studies in children suggest ST59-SCC*mec*IV is the predominant CA-MRSA clone [11]. In addition, there is a worldwide growing spread of the ST398 clone between animals and humans with animal contact or between humans and humans living in animal-free environments [12–14]. Human-associated ST398 is also the most prevalent CA-SA clone in adult SSTIs in China [15].

In our previous study, HA-SA ST239 isolates had improved nasal colonization ability compared with CA-SA ST398 [16]. In addition, the CA-SA ST398 clone had increased virulent ability compared with the HA-SA ST239 clone in the skin infection model [17]. Recent studies have shown the critical adaptation of the HA-MRSA ST239 clone to the highly selective nosocomial setting and its evolution to increased levels of methicillin resistance at the cost of reduced virulence, enhancing its ability to become a successful nosocomial pathogen [18]. The high methicillin resistance of HA-MRSA also attenuates the staphylococcal accessory gene regulator (*agr*)-regulating sensing system, leading to decreased virulence and immobility in the community, whereas the emerging CA-MRSA typically has lower production of penicillin-binding protein 2a (encoded by *mec*A), hence holding its full-scale virulence for successful predominance in the community environment [19].

Transcriptome studies have documented that different *S. aureus* strains show tremendous variations in gene transcriptional profiles causing diverse pathogenesis, despite sharing a core genome with the similarity of approximately 82% [20, 21]. Thus, it is likely that both the presence of multiple mobile genetic elements and temporal alterations in the expression of bacterial core factors contribute to the virulence in *S. aureus*. The ESAT-6 secretion system (Ess) of *S. aureus* exhibits genetic diversity and organizational variation across the species [22]. However, the higher expression of the conserved membrane protein EssB promotes the virulence and facilitates the resistance to innate host defense in the emergent CA-SA clone [17]. In addition, Protein A (SpA), as a cell-wall anchored protein, has been verified to conduce to the higher durative tissue damage in hosts infected by HA-MRSA ST239 compared with those by CA-SA ST398 [16].

However, most studies only suggest one respective prominent factor functioning in the durative damage with HA-SA ST239 infection or in the acute damage with CA-SA ST398 infection. Actually, a regulatory network including multiple factors may cause particular clinical syndromes. A key example of this is based on a previous study showing that the marked hyper-virulence of CA-SA isolates is seemingly due to differential gene expression deriving from the enhanced activity of regulators such as *agr* [23]. Agr up-regulated the expression of many virulence factors such as α-toxin (*hla*), *essB*, etc. [17] and repressed surface adhesins that assist the organism adhere to the host and develop biofilms [23]. These surface adhesins, including microbial surface components recognizing adhesive matrix molecules (MSCRAMMs), some non-covalently attached adhesins, and cell wall teichoic acid, mediate the adhesion to extracellular matrix and plasma proteins [24–28]. Hence, in this study, we sought to characterize the proteome of clinically significant pathogenic strains presently afflicting human beings in the nosocomial and community settings in China. We intended to identify and quantify the production of key network-based factors that enable this pathogen to promptly infect individuals and then cause diseases.

## Methods

### Bacterial strains and growth conditions

Bacteria were identified as staphylococci by classic microbiological methods, including Gram staining and catalase and coagulase activity in rabbit plasma. *S. aureus* strains were further categorized by VITEK2 automated systems (BioMérieux, France). CA-SA was defined as an isolate that was obtained either from an outpatient or from an inpatient ≤ 72 h after hospital admission and without the patient having any of the following risk factors: contact with the hospital environment in the preceding 6 months, residence in a long-term care facility in the preceding 12 months, *S. aureus* infection in the preceding 12 months, presence of a central vascular catheter at the time of infection. HA-SA was defined as an isolate that was obtained from an inpatient > 72 h after hospital admission, or from an outpatient or inpatient ≤ 72 h after hospital admission or having at least one of those risk factors listed above.

The HA-SA ST239 and CA-SA ST398 *S. aureus* isolates used for phenotypic experiments were randomly selected from the clinical isolates with typical infectious manifestations at Shanghai teaching hospitals during 2005–2014. The four HA-SA ST239 and four CA-SA ST398 *S. aureus* strains used for mass spectrometry were selected from the above isolates with relatively moderate phenotypes. The four HA-SA ST239 isolates were all MRSA and caused respiratory infection, while all four CA-SA ST398 isolates caused SSTIs, and only half of them were MRSA. All bacterial strains used in this study were list in Table 1. The isolates were grown in tryptic soy broth (TSB; Oxoid, Basingstoke, Hampshire, UK) at 37 °C with agitation.

**Table 1:** Bacterial strains used in this study

No.	Isolate name	CA/HA	MRSA/MSSA	MLST	Spa type	Infection type
*Strains for mass spectrometry*						
1	Ji95	HA	MRSA	ST239	t037	Respiratory
2	Ji99	HA	MRSA	ST239	t037	Respiratory
3	2011-1046	HA	MRSA	ST239	t037	Respiratory
4	2009-770	HA	MRSA	ST239	t037	Respiratory
9	2009-898	CA	MSSA	ST398	t034	SSTIs
10	2010-191	CA	MRSA	ST398	t571	SSTIs
11	Ji92	CA	MRSA	ST398	t034	SSTIs
12	2005-109	CA	MSSA	ST398	t034	SSTIs
*Strains for RT-PCR, western-blot, adhesion, biofilm and hemolysis assays*						
1	Ji95	HA	MRSA	ST239	t037	Respiratory
2	Ji99	HA	MRSA	ST239	t037	Respiratory
3	2011-1046	HA	MRSA	ST239	t037	Respiratory
4	2009-770	HA	MRSA	ST239	t037	Respiratory
5	2010-1030	HA	MRSA	ST239	t030	SSTIs
6	2012-169	HA	MRSA	ST239	t037	SSTIs
7	2012-32	HA	MRSA	ST239	t037	Respiratory
8*	2012-97	HA	MRSA	ST239	t030	Respiratory
9	2009-898	CA	MSSA	ST398	t034	SSTIs
10	2010-191	CA	MRSA	ST398	t571	SSTIs
11	Ji92	CA	MRSA	ST398	t034	SSTIs
12	2005-109	CA	MSSA	ST398	t034	SSTIs
13	2010-38	CA	MSSA	ST398	t034	SSTIs
14	2005-577	CA	MSSA	ST398	t571	SSTIs
15	2007-524	CA	MSSA	ST398	t034	Respiratory
16*	2007-1089	CA	MSSA	ST398	t034	SSTIs

*No. 8 and No. 16 were not available for RT-PCR assay

### Multi-locus sequence typing (MLST)

MLST was performed as previously described [29]. The PCR amplicons of seven *S. aureus* housekeeping genes (*arcC*, *aroE*, *glpF*, *gmk*, *pta*, *tpi*, and *yqiL)* were obtained from chromosomal DNA. The sequences of the PCR products were compared with the existing sequences available at the MLST website [30], and the allelic number was determined for each sequence.

### *S. aureus* infection of epithelial cells

For the adhesion assay, *S. aureus* was cultured in TSB for 8 h, and the cell pellet was washed twice with F12 K medium. Human epithelial A549 cells were cultured in F12 K medium supplemented with fetal bovine serum (FBS, 10%) and l-glutamine (2 mM) in T75 flasks at 37 °C and 5% CO_2_. Cells were liberated from flasks using trypsin-EDTA solution (Sigma-Aldrich, St Louis, MO, USA), resuspended in culture medium and seeded at 2 × 10^6^ cells/well in a final volume of 500 µl for 120 min at 37 °C with 5% CO_2_. Cells were washed 3 times in F12 K, and 10^8^ CFU *S. aureus* were added and incubated with the cells for 120 min. The coverslips that were used to determine the total number of associated CFU (adherent and internalized) were dip washed three times and subsequently lysed by the addition of 500 μl of 0.1% deoxysodium cholate solution. Bacterial CFU values were enumerated by serial dilutions of epithelial cell lysates and plating onto TSA plates.

### Semi-quantitative biofilm assay

Semi-quantitative biofilm assays were performed as described elsewhere [31]. Subsequently, cells were fixed in Bouin fixative. The fixative was removed after 1 h, and the wells were washed with PBS. Organisms in the wells were then stained with 0.4% (wt/vol) crystal violet, and the floating stain was washed off with slowly running water. After drying, the stained biofilm was read using a MicroELISA autoreader (BioRad) at 570 nm.

### Lysis of erythrocytes by culture filtrates

The supernatants were collected from bacterial cultures grown for 15 h. Hemolytic activities were determined by incubating samples with human red blood cells (2% v/v in Dulbecco’s phosphate-buffered saline, DPBS) for 1 h at 37 °C. Hemolysis was determined by measuring the optical density at 540 nm using an ELISA reader. The assay was performed in triplicate.

### Label-free quantitative proteomic analysis

The overnight cultures were diluted 1:100 in 50 ml of TSB and incubated at 37 °C with shaking at 220 rpm until grown to post-exponential growth phase (8 h) (OD600 ~ 4.0). Samples were normalized according to the OD600 reading (OD600 = 2.0). The cells were collected by centrifugation at 14,000*g* at 4 °C for 15 min. The supernatants were removed and the pellets were washed twice with PBS and then suspended in the lysis buffer of 95 μl 20 mM Tris (pH 8.0) and exposed to 50 μg/ml lysostaphin at 37゜C for 30 min. Cells debris was removed by centrifugation at 14,000*g* for 30 min at 4 °C. Cell lysates were mixed with 25 μl 5X protein loading buffer and boiled for 10 min. The cell particles were removed by centrifugation at 14,000*g* for 5 min at 4 °C. 12% SDS-PAGE was used to pre-fractionate the proteins in the loading volume of 10 μl. The protein samples were subjected to in-gel digestion. Finally, the extracted peptides were vacuum-dried prior to LC-MS/MS analysis using a nanoflow liquid chromatography instrument (EASY-nLC 1000, Thermo Scientific) coupled to an ion trap mass spectrometer (LTQ Velos Pro. Thermo Scientific) in the data-dependent mode. The detailed LC-MS/MS settings have been described elsewhere [32]. The peptide mass peaks of the fractions obtained by LC-MS/MS were compared with the Universal Protein Resource database (UniProt) (http://www.uniprot.org/). The differentially expressed proteins were filtered by the following cutoff criteria: the P value (Mann–Whitney U test) was lower than 0.05, and the fold changes (ST239/ST398) were higher than 1.5-fold or lower than 0.67-fold. The volcano plot for significantly differentially expressed proteins was produced by Excel.

### Hierarchical analysis

Hierarchical analysis of protein expression was performed using MEV (Multi Experiment View) cluster software and the Hierarchical Clustering (HCL) tool [33] selecting the distance metric of Pearson’s correlation and linkage method of average linkage clustering.

### Protein–protein interaction (PPI) network construction

The Search Tool for the Retrieval of Interacting Genes/Proteins (STRING) database [34] identifies the interactions of gene products, including not only the direct physical interactions of proteins but also their functional interactions. To evaluate the interactions among the differentially expressed genes, we uploaded these genes and drew a color-coded protein-protein interaction network graph. We then imported the PPI data in text format into the Cytoscape program [35] to visualize the relationships and used its network analyzer plug-in to analyze the PPI network [35].

### Real-time quantitative reverse transcription-PCR (RT-PCR)

For RNA isolation, cells were collected from bacterial cultures grown to post-exponential growth phase (8 h) and then harvested and washed twice in DEPC water. Cells were disrupted by shaking with a Mini-Beadbeater (Biospec Products) at maximum speed for 30 s. Tubes were then incubated on ice for 5 min and the suspensions were centrifuged. Total RNA was isolated using an RNeasy minikit (Qiagen) as recommended in a standard protocol. Complementary DNA (cDNA) was synthesized from total RNA using the QuantiTect reverse transcription system (Qiagen, Hilden, Germany) according to the manufacturer’s instructions. Oligonucleotide primers were designed using Primer Express (Additional file 1: Table S1). The resulting cDNA and negative control samples were amplified using the QuantiTect SYBR green PCR kit (Qiagen). Reactions were performed in a MicroAmp Optical 96-well reaction plate using a 7500 Sequence Detector (Applied Biosystems, Foster City, CA, USA). Standard curves were determined for each gene using purified chromosomal DNA at concentrations of 0.005–50 ng/ml. All quantitative RT-PCR experiments were performed in duplicate, with *gyrB* as an internal control [36].

### Construction, expression and purification of recombinant fusion protein

The isolates S0385 (GenBank accession number AM990992) and TW20 (GenBank accession number FN433596) respectively served as the reference genomes of CA-SA ST398 and HA-SA ST239 strains. The *sarX* gene was conserved between both of the reference genomes. Genomic DNA extracted from CA-SA ST398 isolate S0385 (GenBank accession number AM990992) served as the PCR template. The *sarX* gene was amplified using the forward primer 5′- CGGGATCCTTGAATACTGAGAAATTAGAAACAT-3′ and the reverse primer 5′-CGGAATTCTTAAATATTTAAAAATTGTTCTACA-3′. The respective PCR product was digested with *BamHI* and *EcoRI*. The PCR product was ligated into pET28a. The resulting plasmid were transformed into *Escherichia coli* Top10 strain. The correct nucleotide sequence was confirmed by sequencing. The resulting construct was transformed into *Escherichia coli* strain BL21 (DE3) for isopropyl-β-D-1-thiogalactopyranoside (IPTG)-induced expression according to the manufacturer’s instructions. His-tagged SarX protein was respectively affinity-purified from cleared lysates with Ni-NTA resin (Qiagen) according to manufacturer’s construction. The protein concentration was determined by the bicinchoninic acid assay (Yeasen Bio, China).

### Production of rabbit antisera and purification of antibodies

Purified proteins (for SarX) or peptides (for AgrA, AgrC, Atl, ClfA, Cna, IsaA, IsaB, LtaS, YycG and FnbpA) were used as an immunogen for the production of rabbit polyclonal antisera (provided by GLbiochem China). The isolates S0385 (GenBank accession number AM990992) and TW20 (GenBank accession number FN433596) also resctively served as the peptide template of CA-SA ST398 and HA-SA ST239 strains. Synthetic peptides corresponding to amino acids of AgrA (DSKERIVYFKNKEHC), AgrC (KYKRNQEEIETYYE), ClfA (SSKEADASENSMTQ), Atl (IGEVGKYFDIPQYK), Cna (TFDDKNGKIQNGDT), IsaA (TMPGWGPTNTVDQQ), IsaB (GKDLKKENGKTKEAD), LtaS (NYTKQRQTEPNPEYY), YycG (EKELLDNFKKNITQ) and FnbpA (ELPETGGEESTNKGM) were synthesized by GLbiochem, China. All peptide fragments were respectively conserved between HA-SA ST239 and CA-SA ST398 strains. The antibody IgG were purified by the protein A affinity column Hitrap rProteinA FF (GE Healthcare) using an AKTA purifier (GE Healthcare) according to the manufacturer’s specifications. Eventually, only polyclonal antibodies for AgrA, Atl and ClfA peptides and SarX protein were successfully obtained.

### Western blot analysis

For protein detection, cells were also collected from cultures of *S. aureus* strains grown to post-exponential growth phase (8 h). Samples were normalized according to the OD600 reading (OD600 = 2). Cells were harvested and resuspended in 50 μl of TE buffer (10 mM Tris-HCl, 1 mM EDTA, pH 8.0) and treated with lysostaphin (50 μg/ml) for 30 min at 37 °C. Samples were mixed with protein loading buffer and boiled for 10 min. Equivalent amounts of proteins were separated on 12% SDS-PAGE gels and were electrotransferred to PVDF membranes (Invitrogen). After blocking, the membranes were incubated with respective antiserum at 4 °C overnight and then were incubated with horseradish peroxidase-conjugated secondary antibody at room temperature for 1 h. To ensure consistency between blots, Sortase A (SrtA) was used as the loading control. The SrtA antibody was kindly donated by Taeok Bae, Indiana University School of Medicine-Northwest, Gary, Indiana, United States of America. All the other antibodies were generated by GLbiochem, China. Images of Western blots were acquired using a Tanon-5200 system. Densitometry analysis was performed with ImageJ software for each protein band in reference to the corresponding SrtA band. All Western blot experiments were performed in duplicate.

### Statistical analysis

The Mann–Whitney U test was performed to analyze the statistical significance of difference in protein expression from mass spectrometry. Student’s t-test was used to analyze the following data resulting from RT-PCR and Western blotting. All data were analyzed using Prism (GraphPad Software, Inc., La Jolla, CA, USA), and *P* values less than 0.05 were deemed statistically significant. The error bars in all graphs show the standard deviation (± SD). The asterisks indicate the following: *P < 0.05, **P < 0.01, ***P < 0.001.

## Results

### HA-SA isolates showed increased epithelial cell adhension ability and biofilm formation compared with CA-SA isolates

**Fig. 1:**
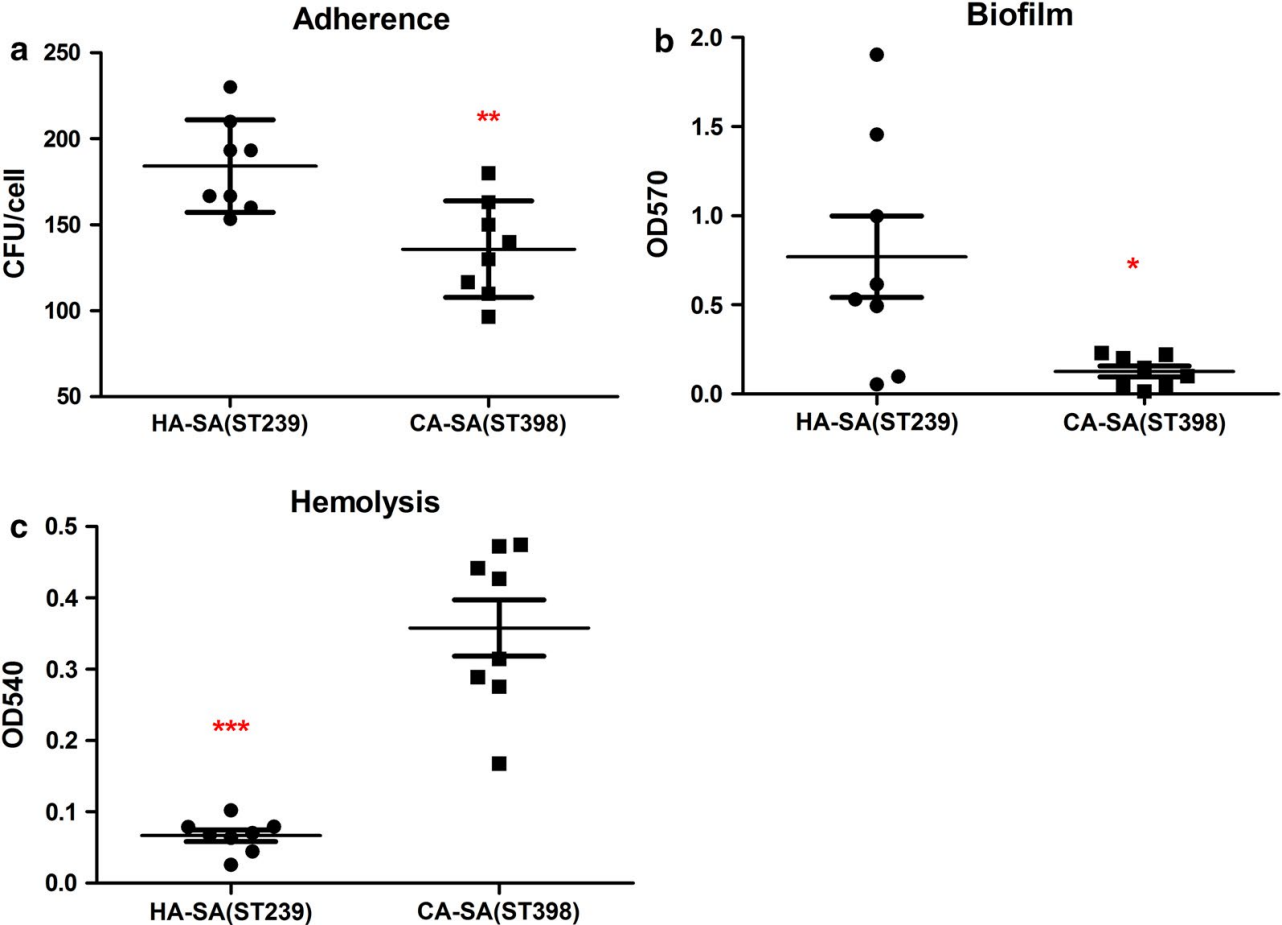
Comparison between CA-SA ST398 and HA-SA ST239 isolates in adhesion, aggregation and virulence characteristics. **a** Colony counts of adhesive and internalized bacteria above in A549 epithelial cells infected for 2 h. A ~ 0.4-fold increase could be seen for HA-SA ST239 isolates compared with that for CA-SA ST398 strains. **b** Biofilm formation. Biofilm formation abilities were calculated by the semiquantitative biofilm assay and were read using a MicroELISA autoreader (BioRad) at 570 nm. An obvious increase could be seen for HA-SA ST239 isolates compared with that for CA-SA ST398 strains. **c** Hemolysis (erythrocyte lysis). Hemolytic activities were determined by incubating culture filtrates with human red blood cells. A significant increase could be seen for CA-SA ST398 isolates compared with that for HA-SA ST239 strains. *P < 0.05, **P < 0.01, ***P < 0.001 (unpaired t-test). The data is representative of three independent experiments

The adhesion and aggregation ability of *S. aureus* is crucial for bacterial colonization, infection and spread in human [37]. We presumed that the differences in transmissibility of HA-SA ST239 and CA-SA ST398 may be a reflection of the expression variation in surface adhesins (e.g., MSCRAMMs) between these strain types. Here, we used human alveolar epithelial cell A549 as a model to test the adhesion ability of ST239 and ST398. We observed significantly increased adherence of HA-SA ST239 isolates compared with CA-SA ST398 isolates (*P* = 0.007) (Fig. 1a). Furthermore, the predominant Chinese HA-SA ST239 lineage was found to have improved biofilm formation ability compared with the CA-SA ST398 strains (Fig. 1b), suggesting that HA-SA would cause more severe intercellular bacterial aggregation. However, the interactive factors facilitating the colonization of HA-SA ST239 were unclear.

### HA-SA isolates had decreased hemolysis ability compared with CA-SA isolates

α-toxin (Hla) can lyse red cells and is one of the most essential virulence factors in *S. aureus* [38]. To test the expression of Hla in the culture supernatant, the cytolytic potential was measured by analyzing the lysis of human erythrocytes. Our results showed that the hemolysis activity of CA-SA ST398 isolates strongly exceed that of HA-SA ST239 isolates (Fig. 1c), thus improving the more acute infection of CA-SA than HA-SA. Nonetheless, the interactive factors promoting the higher virulence of CA-SA ST398 remained to be determined

### Proteomic analysis

**Fig. 2:**
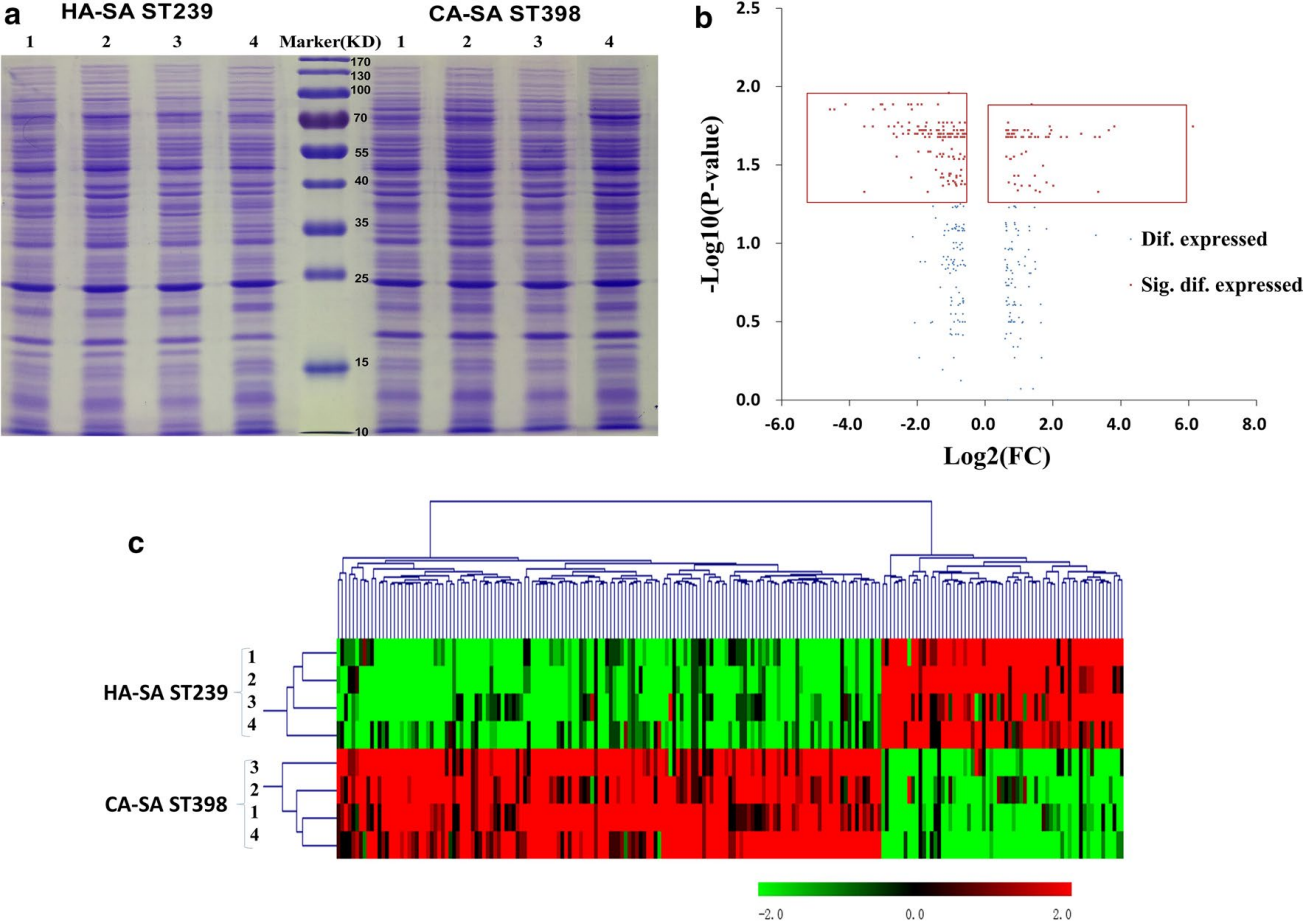
**a** Comparison of the total proteins between the CA-SA ST398 and HA-SA ST239 isolates by SDS-PAGE. **b** Volcano plot of differentially expressed proteins in the HA-SA ST239 versus CA-SA ST398 isolates. **c** Hierarchical cluster analysis was conducted for all the differentially expressed proteins in the HA-SA ST239 group versus the CA-SA ST398 group

SDS-PAGE analysis was performed on bacteria cells for four CA-SA ST398 isolates and four HA-SA ST239 clinical isolates at the post-exponential phase of growth (8 h). We first confirmed the comparable protein bands within four CA-SA ST398 isolates or within four HA-SA ST239 isolates (Fig. 2a). Then, Spectral counting based on LC-MS/MS proteomics was performed to investigate the difference in the protein production between four CA-SA ST398 clinical isolates and four HA-SA ST239 clinical isolates. The CA-SA ST398 group was compared with the HA-SA ST239 group to identify differentially expressed proteins. Figure 2b showed the volcano plot for the differentially expressed proteins in the ST239 and ST398 groups. These proteins with a P value by Student’s t-test lower than 0.05 and with an ST239/ST398 change higher than 1.5-fold or lower than 0.67-fold were considered as differentially expressed proteins. All the 209 differentially expressed proteins (64 up-regulated in the HA-SA ST239 group and 145 up-regulated in the CA-SA ST398 group) were listed in Tables 2 and 3 and the detailed information on differentially expressed proteins in each isolate were depicted in Additional file 1: Tables S2 and S3.

**Table 2:** All up-regulated proteins in the HA-SA ST239 group

Uniprot acc no.	Prot description	FC* (239/398)	P value
*Known virulence*-*related factors*			
D2NA85	FnbpA**	13.99	0.02
D2N8V1	Aerolysin/Leukocidin family protein	7.10	0.02
D2N8V0	Synergohymenotropic toxin	5.30	0.02
D2N3G2	***SpA***	3.26	0.03
D2NAS2	***Cna***	2.54	0.02
D2N627	***Atl***	2.46	0.02
D2N5J4	***ClfA***	2.11	0.02
*Staphylococcal antigen*			
D2N9N2	***SsaA***	4.70	0.02
D2NAF1	***IsaA***	2.50	0.04
D2NAM0	***IsaB***	1.73	0.02
*Other known proteins*			
D2N382	Penicillin-binding protein 3 (Pbp 3) (Pspb20)	69.88	0.02
D2N4Y0	Acetyl-CoA acetyltransferase	12.51	0.02
D2NAM6	N-acetylmuramoyl-l-alanine amidase	10.41	0.02
D2N4M0	N-acetylmuramoyl-l-alanine amidase Sle1	9.41	0.02
D2N5C6	***LtaS***	4.03	0.04
D2N9E7	HAD-superfamily hydrolase, subfamily IIB	3.87	0.02
D2N751	MsrA	3.75	0.02
D2NAT9	MnmE	3.74	0.02
D2N835	Universal stress protein family, putative	3.66	0.02
D2N6J2	Sun	3.51	0.04
D2N9E2	Morphine 6-dehydrogenase (Naloxone reductase)	3.46	0.02
D2NAH7	PanD	3.05	0.05
D2N655	YwbD	2.95	0.02
D2NAK8	Alkaline phosphatase 3 (Alkaline phosphataseIII) (APase III)	2.80	0.05
D2NAM5	Phage infection protein	2.77	0.02
D2N7Y0	RuvB	2.75	0.04
D2N917	Cardiolipin synthetase (Cardiolipin synthase) (CLsynthase)	2.58	0.01
D2N7V5	UPF0473 protein	2.58	0.02
D2N410	5 ~-nucleotidase, lipoprotein e (P4) family	2.47	0.04
D2N9P4	Inositol monophosphatase family protein	2.40	0.02
D2N6X6	Glycine betaine transporter OpuD	2.31	0.03
D2N6I0	PhnB	2.24	0.02
D2N953	Putative transcriptional regulator superfamily	2.10	0.02
D2N755	YphP	2.10	0.04
D2N5A8	NagA	2.10	0.03
D2N7I6	ScpB	1.97	0.04
D2N750	MsrB	1.94	0.05
D2N657	Phosphocarrier protein HPr	1.92	0.02
D2N6I4	RpoZ	1.84	0.04
D2N9L8	Butyryl-CoA dehydrogenase	1.84	0.02
D2N6L2	UPF0122 protein	1.81	0.04
D2N7K7	Lipoamide acyltransferase component of branched-chain alpha-keto aciddehydrogenase complex	1.80	0.02
D2N6J3	RlmN	1.80	0.03
D2N681	Inositol-1-monophosphatase (IMPase) (Inositol-1-phosphatase) (I-1-Pase)	1.76	0.02
D2N5S9	AddA	1.73	0.02
D2N5P1	HAD-superfamily subfamily IIA hydrolase	1.69	0.03
D2N7P4	Superoxide dismutase	1.68	0.02
D2N7T3	RsfS	1.66	0.03
D2N842	Tpx	1.64	0.02
D2N7J2	Oxidoreductase	1.60	0.02
D2N941	UDP-N-acetylglucosamine 2-epimerase	1.60	0.02
D2N7L6	NusB	1.58	0.02
D2N6K8	AcpP	1.58	0.02
D2N4I9	HsdM	1.58	0.02
D2N4U4	RplJ	1.55	0.02
D2N6F6	MurD	1.54	0.02
D2N5D9	Ribonucleoside-diphosphate reductase, beta subunit	1.54	0.02
D2N8L2	Aminopeptidase PepS	1.54	0.03
*Unknown proteins*			
D2N9M8	Uncharacterized protein	3.63	0.02
D2N5P0	Uncharacterized protein	2.49	0.04
D2N4W0	Uncharacterized protein	2.17	0.02
D2NA92	Uncharacterized protein	1.85	0.02
D2N685	Uncharacterized protein	1.62	0.04
D2N588	Uncharacterized protein	1.61	0.02

*The calculation of FC (239/398) in this table is dividing the average value of peptide mass of proteins in ST239 strains by that average value in ST398 strains

**Bold italics in this table is used to highlight the selected significantly differentially expressed genes for HA-SA ST239 up-regulated network construction

**Table 3:** All up-regulated proteins in the CA-SA ST398 group

Uniprot acc no.	Prot_description	FC* (398/239)	P value
*Known virulence*-*related factors*			
D2N8W5	AgrC**	23.91	0.01
D2N8W6	***AgrA***	7.20	0.02
D2N7I3	***SrrB***	2.02	0.02
D2N8Z3	***SigB***	1.98	0.03
D2N718	Conserved virulence factor B (SAPIG1392)	1.98	0.02
D2N5H5	***ClpP***	1.77	0.02
*Transcriptional regulator*			
D2N576	***SarX***	9.91	0.01
D2N9W6	AraC family regulatory protein	4.48	0.01
D2N9P8	Phosphosugar-binding transcriptional regulator, RpiR family	3.99	0.02
D2N3I9	Transcriptional regulator, GntR family	3.32	0.02
D2N9Q7	Phosphosugar-binding transcriptional regulator	3.07	0.03
D2N823	***PhoP***	2.36	0.02
D2N6S6	Glycerol uptake operon antiterminator regulatory protein	2.41	0.03
D2N8K6	RecX	2.08	0.04
*Arginine and proline metabolism*			
D2NAL7	ArcA	6.11	0.03
D2NAL6	ArcB	4.18	0.02
D2NAL4	ArcC	2.52	0.03
*Multidrug resistance*			
D2N9T7	Multidrug resistance protein A	4.72	0.02
D2N552	Multidrug resistance ABC transporter ATP-binding and permease protein	3.84	0.02
*Two*-*component system*			
D2N9Y1	NarH	2.36	0.03
D2N361	***YycG***	1.89	0.03
*Pyrimidine metabolism*			
D2NAJ6	NrdD	11.84	0.02
D2N6H8	PyrE	2.36	0.02
D2N6H3	PyrB	2.11	0.04
D2N942	Upp	1.52	0.02
*Pyruvate metabolism*			
D2N3S5	PflB	2.37	0.04
D2N9V0	Mqo	2.26	0.02
D2N7L8	AccC	1.78	0.02
*Histidine metabolism*			
D2N9R5	HutU	17.44	0.01
D2N9R4	HutI	3.59	0.02
D2N9R7	HutG	1.82	0.04
*Glycolysis/gluconeogenesis*			
D2NAA0	Fbp	2.63	0.02
D2N9Z9	GpmA	2.50	0.02
D2N8B5	PckA	2.34	0.02
*Other known proteins*			
D2N3K5	CapC	21.85	0.01
D2N3F9	Antigen, 67 kDa	11.84	0.05
D2N5J2	Probable membrane protein	8.22	0.01
D2NA54	3-oxoacyl-[acyl-carrier-protein] reductase (3-ketoacyl-acyl carrier protein reductase)	7.79	0.01
D2N5B9	QueC	6.59	0.01
D2N3D6	Amidohydrolase 2	6.51	0.02
D2N543	Iron dependent repressor	6.42	0.02
D2N8W0	Nitroreductase family protein	6.12	0.02
D2N628	Acetyl transferase	5.96	0.02
D2NA36	Para-nitrobenzyl esterase	5.36	0.02
D2N4R9	FolP	4.83	0.02
D2N3T8	ABC transporter, substrate-binding protein	4.82	0.02
D2N549	Teichoic acid biosynthesis protein X	4.81	0.01
D2N944	UPF0340 protein	4.72	0.02
D2N4D0	Stage 0 sporulation protein J	4.54	0.01
D2N5Q4	Cytosol aminopeptidase family protein	4.53	0.04
D2N4P0	Stage 0 sporulation protein YaaT	3.98	0.02
D2N526	Hydrolase, alpha/beta hydrolase fold family	3.92	0.02
D2N723	DapB	3.80	0.02
D2N3Q0	Pts system eiibc component	3.71	0.02
D2N8G9	ABC transporter EcsB	3.67	0.01
D2N3F6	Aminoacylase	3.58	0.02
D2N585	Lipoprotein, putative	3.47	0.02
D2N6B0	SpoU rRNA Methylase family protein	3.42	0.02
D2N349	NnrD	3.39	0.02
D2NAA6	Glyoxalase family protein	3.37	0.02
D2N362	YycH protein	3.21	0.03
D2N6F0	UPF0747 protein	3.18	0.02
D2N3K2	Aldehyde-alcohol dehydrogenase 2	3.12	0.02
D2N9Q3	Phosphoglycolate phosphatase	3.04	0.02
D2N411	ABC transporter, permease protein	3.01	0.02
D2N7J1	Asppase	2.69	0.03
D2N7R6	NfeD	2.63	0.01
D2N4E3	Phosphoglycerate mutase family protein	2.59	0.02
D2N7R2	PhoH family protein	2.55	0.02
D2N5I8	Rnr	2.52	0.02
D2N7Z5	Tag	2.48	0.02
D2N9Z1	Zinc-binding lipoprotein AdcA	2.47	0.04
D2N4S0	FolB	2.41	0.04
D2N363	YycI protein	2.39	0.02
D2NA39	ABC transporter ATP-binding protein	2.36	0.04
D2N499	NAD-dependent epimerase/dehydratase	2.36	0.02
D2N6R1	PgsA	2.34	0.02
D2N5Y1	NadK	2.34	0.02
D2N804	EngB	2.18	0.02
D2N421	NanE	2.15	0.04
D2N6C3	MurI	2.12	0.02
D2N356	DHH subfamily 1 protein	2.11	0.02
D2N8M1	Map	2.10	0.02
D2N5H2	UPF0042 nucleotide-binding protein	2.10	0.03
D2N7R9	Ribosomal RNA small subunit methyltransferase E	2.10	0.01
D2N870	Ftsk/spoiiie family protein	2.09	0.02
D2N9Y2	Nitrate reductase, alpha subunit	2.04	0.02
D2N5V8	FabH	1.95	0.02
D2NA13	ABC transporter, ATP-binding/permease protein	1.92	0.04
D2N6V0	Cardiolipin synthetase (Cardiolipin synthase) (CLsynthase)	1.92	0.03
D2N6W2	Catalase	1.90	0.02
D2N8Q5	Aldehyde dehydrogenase	1.87	0.02
D2N558	DhaK	1.86	0.04
D2N9V4	TpgX protein	1.84	0.02
D2N860	Fhs	1.82	0.02
D2N8R8	Acyl-coenzyme A:6-aminopenicillanic acid acyl-transferase	1.80	0.04
D2N5D0	ABC transporter permease protein	1.80	0.04
D2N7M6	GcvPB	1.80	0.04
D2N694	Glycerophosphoryl diester phosphodiesterase	1.79	0.02
D2N634	QoxB	1.78	0.02
D2N4Q0	IspE	1.78	0.02
D2N727	LysA	1.77	0.02
D2N6R4	Rny	1.74	0.02
D2N9L2	ModA	1.71	0.03
D2N5F5	DegV family protein	1.69	0.02
D2N520	Iron-binding protein	1.66	0.04
D2N9C8	Alcohol dehydrogenase, zinc-binding domain protein	1.64	0.02
D2N359	PurA	1.64	0.04
D2N6G7	IleS	1.62	0.02
D2N9V2	TagF domain protein	1.61	0.02
D2N6Y5	Transcription antiterminator	1.60	0.04
D2N7P9	ATP-dependent RNA helicase	1.60	0.02
D2N4Q1	PurR	1.58	0.02
D2N5F8	Ribosomal subunit interface protein	1.57	0.02
D2N662	Potassium uptake protein TrkA	1.57	0.02
D2N9C5	Conserved domain protein	1.57	0.02
D2N7J7	Zwf	1.56	0.03
D2N3I0	Acetoin(Diacetyl) reductase (Acetoin dehydrogenase)	1.55	0.02
D2N780	AsnS	1.53	0.02
D2N8B2	2,5-diketo-D-gluconic acid reductase A	1.52	0.02
D2N5Q3	YumB	1.52	0.02
D2N6S5	MutL	1.52	0.03
D2N6M8	TopA	1.52	0.04
D2N523	Hydrolase, alpha/beta hydrolase fold family	1.51	0.02
D2N4S2	LysS	1.51	0.02
*Unknown proteins*			
D2N9S7	Uncharacterized protein	9.98	0.02
D2N965	Uncharacterized protein	8.45	0.01
D2N3Y5	Uncharacterized protein	5.51	0.02
D2N899	Uncharacterized protein	4.17	0.02
D2N9W5	Uncharacterized protein	3.98	0.01
D2N4F3	Uncharacterized protein	3.92	0.03
D2N692	Uncharacterized protein	3.24	0.05
D2N5Y6	Uncharacterized protein	2.69	0.02
D2N5H8	Uncharacterized protein	2.65	0.02
D2N8E4	Uncharacterized protein	2.60	0.02
D2N678	Uncharacterized protein	2.58	0.02
D2N6V3	Uncharacterized protein	2.15	0.02
D2N9T5	Uncharacterized protein	2.07	0.04
D2N8T9	Uncharacterized protein	2.03	0.04
D2N8R7	Uncharacterized protein	1.89	0.02
D2N8B3	Uncharacterized protein	1.65	0.02
D2N6R0	Uncharacterized protein	1.64	0.03
D2N9C6	Uncharacterized protein	1.58	0.02
D2N971	Uncharacterized protein	1.56	0.02
D2N5K3	Uncharacterized protein	1.67	0.03

*The calculation of FC (398/239) in this table is dividing the average value of peptide mass of proteins in ST398 strains by that average value in ST239 strains

**Bold italics in this table is used to highlight the selected significantly differentially expressed genes for CA-SA ST398 up-regulated network construction

Hierarchical cluster analysis was further performed for all the differentially expressed proteins in the HA-SA ST239 group versus the CA-SA ST398 group since HCL method allows samples that are highly similar in quantitative profiles to be merged in an agglomerative fashion. As shown in Fig. 2c, we were able to distinguish the two different types of clinical SA by identifying those differentially expressed proteins. Although there might be some exceptions of individual isolate whose expression was not in accordance with the whole expression pattern of the certain protein, it won’t influence the clustering of the four HA-SA ST239 strains or the four CA-SA ST398 strains. The results suggested that HA-SA ST239 elicits a much different proteomic profile from CA-SA ST398. To decipher which proteins were related to the respective virulent effect between HA-SA ST239 and CA-SA ST398, string analysis was performed focusing on the network of proteins possibly involved in the pathogenesis of *S. aureus*.

### Interaction network construction and network analysis

**Fig. 3:**
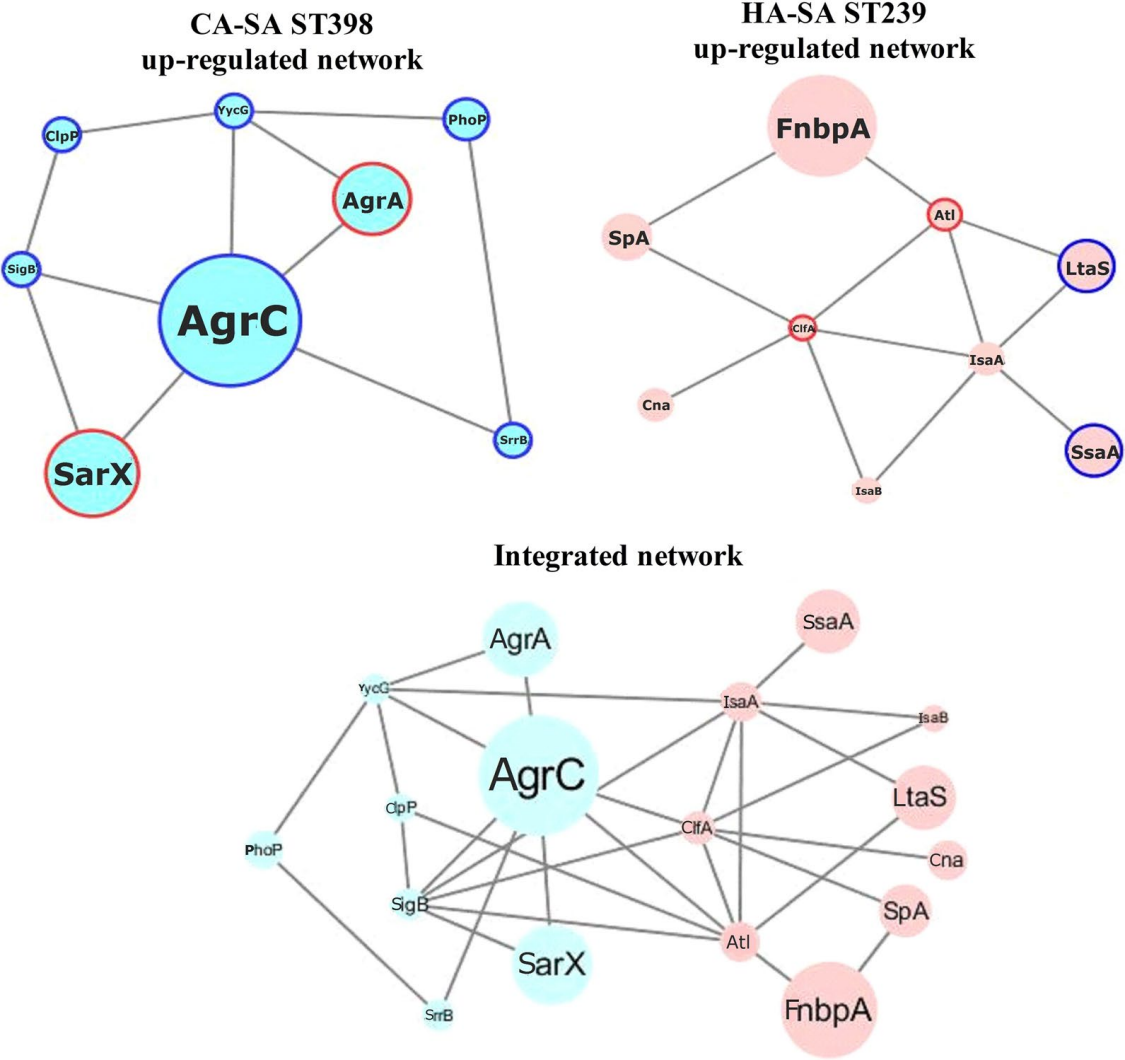
Cytoscape network reconstruction. Circle nodes represent genes. For the genes, the borders of the nodes represent the type of gene regulation determined by LC-MS/MS. The centers of the nodes indicate the gene expression changes; the diameter of the circle is proportional to the level of regulation. The blue outer circles of the nodes depict corresponding results from LC-MS/MS and qRT-PCR analyses (Fig. 4), and the red outer circles indicate corresponding results from LC-MS/MS and Western blot analysis (Fig. 5)

We respectively evaluated 64 up-regulated genes in the HA-SA ST239 group and 145 up-regulated genes in the CA-SA ST398 group using the STRING (version 10.0) database to identify the potential interactions among these gene products. However, the network incorporated numerous isolated nodes and pair-linked nodes providing useless information. Hence, we cut out the differentially expressed genes corresponding to such types of nodes and identified 22 up-regulated proteins in HA-SA group and 95 up-regulated proteins in CA-SA group based on the potential networks (Additional file 2: Figure 1A and B). Next, a PPI network was constructed via Cytoscape software to judge the importance order of the differentially expressed genes between HA-SA ST239 and CA-SA ST398. We also combined the functional enrichment in the network and category according to the degree of knowledge of those proteins. The final HA-SA ST239 up-regulated protein network was constituted of 9 nodes and 12 edges, with one as the minimum degree of connectivity of a node and five as the maximum. For the 9 nodes in the network, the average degree of connectivity was 2.67, and the PPI enrichment P value was 7.22e^−08^. While the resulting CA-SA ST398 up-regulated protein network was composed 8 nodes and 11 edges, and the average degree of connectivity for the 8 nodes was 2.75, the PPI enrichment P value was 4.04e^−09^. Meanwhile, the KEGG enrichment analysis of the STRING database showed that there is only one significantly enriched pathway (extracellular region) in the HA-SA ST239 up-regulated network, including 9 proteins—Fibronectin-binding protein A (FnbpA), Immunoglobulin G binding SpA, Bifunctional autolysin (Atl), Clumping factor A (ClfA), Immunodominant staphylococcal antigen A (IsaA), Immunodominant staphylococcal antigen B (IsaB), Lipoteichoic acid synthase (LtaS), Staphylococcal secretory antigen A (SsaA) and Collagen adhesin (Cna)—and also only one significantly enriched pathway (two-component system) in the CA-SA ST398 up-regulated network including five proteins—Accessory gene regulator A (AgrA), Accessory gene regulator C (AgrC), Alkaline phosphatase synthesis transcriptional regulatory protein (PhoP), Staphylococcal respiratory response protein (SrrB), and Sensor histidine kinase (YycG, also named WalK) (Table 4). Based on the knowledge of proteins and their link in the enriched pathways, Staphylococcal accessory regulator X (SarX), RNA polymerase sigma factor B (SigB) and ATP-dependent Clp protease proteolytic subunit P (ClpP) were also identified as candidate key proteins in the CA-SA ST398 up-regulated network. Finally, nine proteins (FnbpA, SpA, Atl, ClfA, IsaA, IsaB, LtaS, SsaA and Cna) and eight proteins (AgrA, AgrC, PhoP, SrrB, YycG, SarX, SigB and ClpP) were, respectively, used for the model reconstruction of the HA-SA ST239 and CA-SA ST398 key virulent network alone and combined (Fig. 3).

**Table 4:** KEGG-enrichment pathway and genes from String analysis

#Pathway ID	Pathway description	Gene count	False discovery rate	Matching proteins in your network (labels)
*Up-regulated in ST239*				
5576	Extracellular region	9	8.08E−05	FnbpA, SpA, Atl, ClfA, IsaA, IsaB, LtaS, SsaA, Cna
*Up-regulated in ST398*				
2020	Two-component system	5	3.27E−05	AgrA, AgrC, PhoP, SrrB, YycG

One node would be considered more significant than the other nodes and would be called the central node when the degree of connectivity of the node was much greater than the average. The central node of a PPI network also represents the core protein in the network. In the current study, all nodes were analyzed through a network analyzer, according to the degree of connectivity of each node which was set as ≥ the mean value to identify candidate key virulent genes. Based on the degree of connectivity of a node, the HA-SA ST239 up-regulated proteins (ClfA, Atl and IsaA) and CA-SA ST398 up-regulated proteins (AgrC, YycG and SigB) were identified as key proteins in the respective network. However, according to the results of the relatively higher level of differentially expression (the fold changes of ST239/ST398 were higher than 4.0-fold or lower than 0.25-fold), the HA-SA ST239 upregulated proteins (FnbpA, LtaS and SsaA) and CA-SA ST398 up-regulaed proteins (AgrA, AgrC and SarX) were identified as crucial candidate proteins. Nevertheless, the IsaA, AgrC, YycG, SigB, FnbpA, LtaS and SsaA could not been verified successfully in the translational level for the lack of effective antibodies. Thus, we selected these four proteins (ClfA, Atl, AgrA, SarX) for further validation of Western blotting.

### Validation of the proteomic data using RT-PCR

**Fig. 4:**
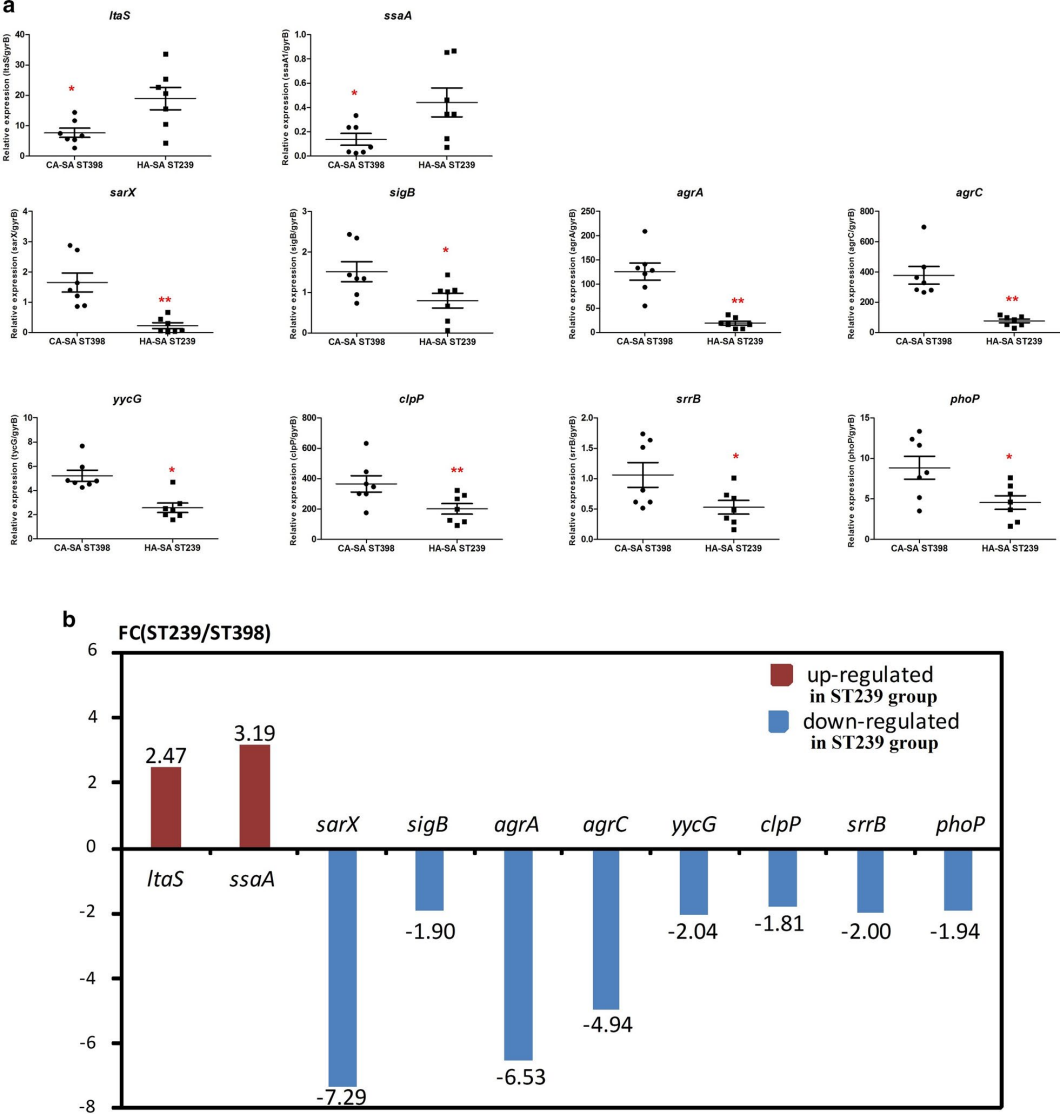
RT-PCR validation. **a** Transcriptional expression levels of the 10 significantly key differentially expressed genes between HA-SA and CA-SA by qRT-PCR in cultures grown to the post-exponential growth phase (8 h) from 17 candidate key differentially expressed genes. *P < 0.05, **P < 0.01, ***P < 0.001 (unpaired t-test). The data is representative of three independent experiments. **b** FC (ST239/ST398) in this figure shows the fold change of the average transcriptionally expressed value of specific gene within HA-SA ST239 strains relative to that within CA-SA ST398 strains. Therefore, Down-regulated in ST239 group equivalents to Up-regulated in ST398 group

The transcription rates of 17 candidate key genes coding for proteins with different expression between HA-SA ST239 group and CA-SA ST398 group detected by LC-MS/MS were tested by RT-PCR. This validation finally selected ten genes with significantly different transcriptional expression between the two groups. As shown in Fig. 4a, the transcriptional levels of eight genes (*sarX*, *sigB, agrA*, *agrC*, *yycG*, *clpP, srrB*, and *phoP*) were significantly greater in the CA-SA ST398 group than in the HA-SA ST239 group, consistent with the proteomic results. However, among the nine proteins (FnbpA, SpA, Atl, ClfA, IsaA, IsaB, LtaS, SsaA and Cna) which were detected up-regulated in the HA-SA ST239 group, only two genes (*ltaS* and *ssaA*) displayed higher transcriptional level in this group, indicating there could be possible differences in translational or post-translational level between ST239 and ST398. As indicated in Fig. 4b showing the fold change of the average transcriptionally expressed value of specific gene within HA-SA ST239 strains relative to the corresponding value within CA-SA ST398 strains, the greatest differentially expressed genes in the transcriptional level were *sarX* and *agrA* (respectively 7.29 and 6.53-fold in ST398 strains compared to ST239 strains).

### Validation of the proteomic data using western blot

**Fig. 5:**
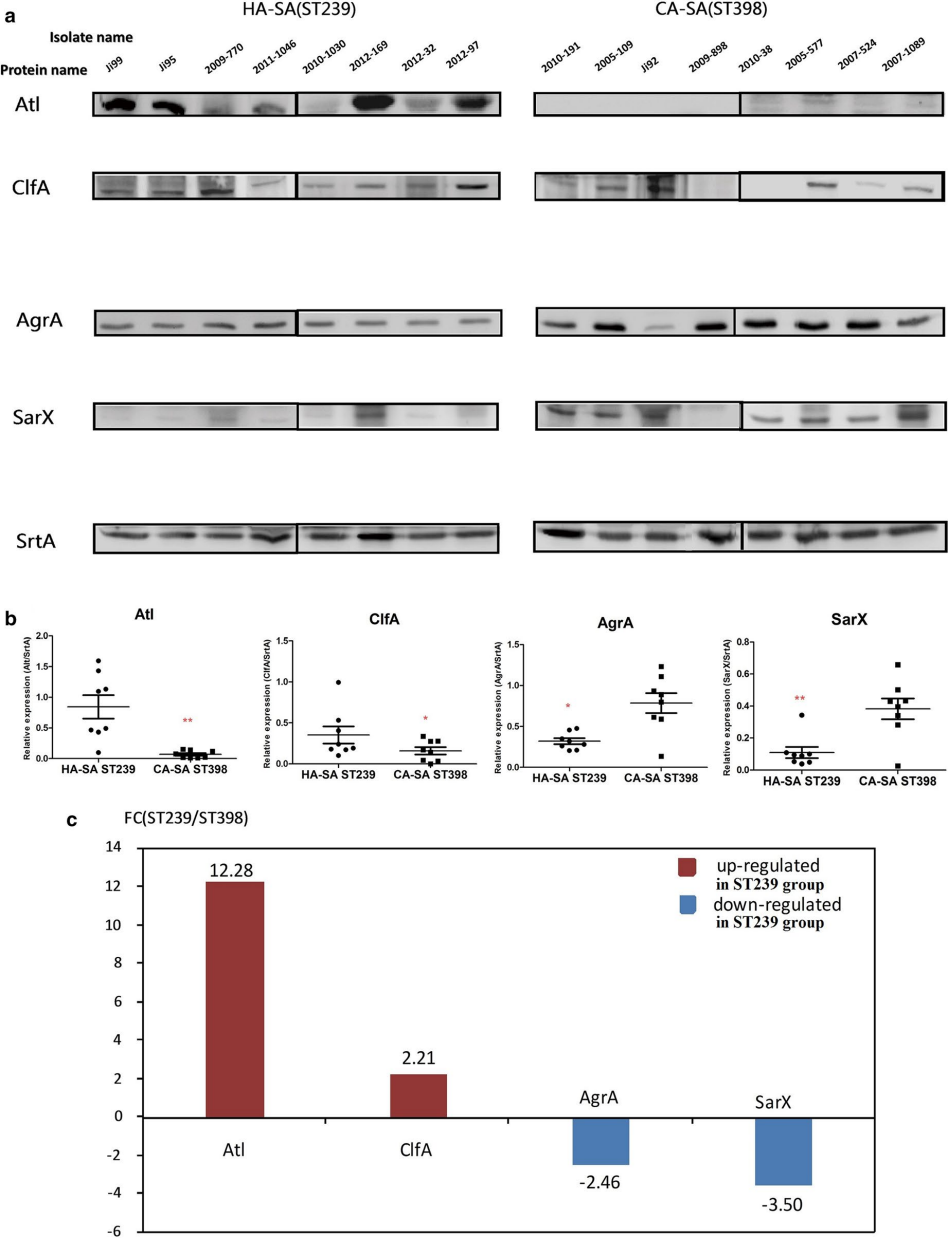
Western blot validation. **a** The expression levels of the four key differentially expressed proteins between HA-SA and CA-SA by densitometry of Western Blot in post-exponential phase cultures (8 h) using sortase A as control. **b** Densitometry plot of Western Blot. The values are normalized versus sortase A signals obtained using the same samples. *P < 0.05, **P < 0.01, ***P < 0.001 (unpaired t-test). **c** FC (ST239/ST398) in this figure shows the fold change of the average translationally expressed value of specific protein within HA-SA ST239 strains relative to that within CA-SA ST398 strains. Therefore, Down-regulated in ST239 group equivalents to Up-regulated in ST398 group

To further confirm the proteomic data, we tested the expression of several proteins by Western blot. In the Western blotting analysis indicated in Fig. 5a, b, the translational expression levels of AgrA and SarX were significantly greater in the CA-SA ST398 group than in the HA-SA ST239 group (*P* = 0.013 and *P* = 0.006 respectively), nonetheless the translational expression levels of Atl and ClfA were significantly higher in the HA-SA ST239 group than in the CA-SA ST398 group (*P* = 0.005 and *P* = 0.048 respectively), which is consistent with the findings in proteomic analysis. Figure 5c showed the fold change of the average translationally expressed value of specific protein within HA-SA ST239 strains relative to the corresponding value within CA-SA ST398 strains. Among the above proteins, Atl was the most differentially expressed protein on the translational level (12.28-fold in ST239 strains compared to ST398 strains).

## Discussion

The study was initiated to identify the key virulent network of HA- and CA-SA. We presented the different phenotypes between HA-SA ST239 and CA-SA ST398. HA-SA ST239 showed more adhesion ability to human alveolar epithelial cells and biofilm than CA-SA ST398 isolates, while the CA-SA ST398 exhibited stronger hemolysis ability than the HA-SA ST239. Further proteomic profile analysis showed that higher expression of surface-related proteins (FnbpA, SpA, Atl, ClfA, IsaA, IsaB, LtaS, SsaA and Cna) may involve in the durative infection in HA-SA ST239, while the higher expression of Agr system (AgrA and AgrC) and its interactive factors (PhoP, SrrB, YycG, SarX, SigB and ClpP) contribute to the higher virulence in CA-ST ST398.

It had been reported that CA-MRSA typically express low quantities of penicillin -binding protein 2a to sustain higher virulence and further predominate in the community setting [19]. Moreover, HA-MRSA strains are less virulent despite the high expression of *mecA* gene [19]. In our study, we set out to profile the proteome of certain clinically significant and relevant strains of SA to discover distinct sets of interactive factors contributing respectively to enhance the virulence of two SA types and cause their different clinical syndromes. Specifically, HA-SA ST239 and CA-SA ST398 representative clinical isolates were analyzed by quantitative methods at the post-exponential phase of growth (8 h), during which the toxins are initially produced and gradually accumulated in a large quantity. We conducted a high throughout and quantitative proteomic analysis to search for the virulent network of HA- and CA-SA [32].

When comparing HA-SA ST239 to CA-SA ST398 during the post-exponential phase of growth, we found that HA-SA ST239 strains significantly expressed a broad range of cell surface-associated virulence proteins (FnbpA, SpA, Atl, ClfA, IsaA, IsaB, LtaS, SsaA and Cna) at a higher level than ST398 strains. The cell surface proteins including MSCRAMMs such as FnbpA, SpA, ClfA and Cna played important roles in the adhesion, biofilm formation and eliciting of endovascular, bone and joint and prosthetic-device infections [39]. For example, FnbpA is involved in bacterial adhesion to host tissues by binding to fibronectin, and to affect biofilm formation [40, 41]. IsaA, involved in peptidoglycan hydrolysis, has been demonstrated as a virulence factor which existed both in the culture supernatant and the cell wall fraction [42]. Furthermore, the inactivation of *isaA* results in the up-regulation of *ssaA*, whose gene product has been demonstrated to own peptidoglycan hydrolase activity and is supposed to be crucial in biofilm formation [43, 44]. These surface proteins play a critical role in preventing the organism from recognition by the host immune system [45]. SpA could adhere to the Fc portion of IgG and Fab fragment of VH3-type B cell receptors, leading to bacterial immune evasion from the host [41, 46]. ClfA factor is an important staphylococcal adhesin, which could bind to dimeric host Fg complex via the carboxy-terminal component of the Fg gamma chain, thus causing staphylococcal accumulation in plasma or purified Fg [47]. As the major Fg binding protein, ClfA also promotes the adherence to immovable Fg- or fibrin-coated surfaces, facilitating its binding to both of blood clots and biomaterials [47]. FnbpA has been shown to be involved in adherence to host cells and to promote internalization by host cells [48, 49]. FnbpA has also been demonstrated to be important in *in vivo* infection by *S. aureus* [50]. The collagen (Cn)-binding protein Cna is a prototype of the MSCRAMMs that plays an important role in staphylococcal pathogenesis both as an adherence factor and as an immune evasion factor. Cna is a proven virulence factor in septic arthritis, where the strength of adhesion to collagen correlates with disease pathogenesis [51, 52]. IsaB, also as a cell-wall associated and secreted virulence factor, could trigger an immune responsive process during the life-threatening septicemia [53]. Through the analysis of microarray data, *isaB* production was also shown to increase in response to some certain settings, such as the existence of biofilms, neutrophil exposure, anaerobic condition and further internalization into human epithelial cells, indicating an important role in infection despite of its involvement in immune escape [54, 55]. Previous study has identified the importance of the *atl* gene products in the initial stages of biofilm formation [56]. Deletion of *atl* whether in *S. aureus* or *S. epidermidis* would cause extensive cell aggregation and following biofilm-negative phenotype [56]. This change of phenotype already indicates that Atl is a necessary factor for the successful partitioning of daughter cells after their division. LtaS participates in the synthesis of lipoteichoic acid (LTA), which is one of the major components of the cell wall in *S. aureus* [57]. As for the observation of increased protein levels of ClfA and Atl in ST239 isolates which was not confirmed by the transcriptional studies, two possible reasons might be responsible for the discrepancy between the transcriptional and translational level. On one hand, regulation of post-transcription, translation, transcript degradation and protein degradation could contribute as much to variation in the final protein concentration. On the other hand, there could be a significant amount of noise and error in mRNA experiment, limiting our access to get a consistent result with protein experiment [58]. Apart from the discrepancies as above, ClfA/Atl/IsaA seemingly acts as central nodes in HA-SA ST239 up-regulated network. However, only the significantly increased expression of ClfA/Atl in HA-SA ST239 group could be verified at the translational level. One would speculate that ClfA/Atl might act as a group leader to gather other team members (SpA, Cna, FnbpA, SsaA, IsaA, IsaB and LtaS) together, thus probably contributing to biofilm formation.

In addition, we found that 8 Agr-interactive network proteins (AgrA, AgrC, PhoP, SrrB, YycG, SarX, SigB and ClpP) are generally up-regulated in the CA-SA strain ST398. This finding is in accord with that the reported transcriptomic studies suggesting a hyper-vital *agr* in CA-SA lineage compared with those in HA-SA lineage [59]. In this study, we have strikingly shown that AgrC in the PPI network reconstruction acted as central node in the ST398 up-regulated network. The higher transcripts of both *agrA* and *agrC* and the higher translational proteins of AgrA were all confirmed in CA-SA ST398. It could be speculated that AgrAC might interact with other connected factors such as PhoP, SrrB, YycG, SarX, SigB and ClpP, however, the regulatory mechanism between them was not clear at present.

Although as the sensor of the *agr* locus, AgrC unexpectedly displayed a higher degree of connectivity to other factors in this bioinformatic analysis than the response regulator AgrA. One possible reason could be the strict selection rule of the significantly differentially expressed proteins for constructing the interaction networks. Some genes/proteins that were regulated by AgrA might be eliminated from the list for the network construction due the cutoff of a P value by Student’s t-test lower than 0.05 and with an ST239/ST398 change higher than 1.5-fold or lower than 0.67-fold. There were links between AgrC and five factors in the construction model (AgrA, SarX, SigB, SrrB and YycG). It was well known that AgrA/AgrC act together, however, other interactions were not so clear. As a SarA paralog, the HTH-type transcriptional regulator (SarX) was initially identified in *S. aureus* by Manna and Cheung (2006). They have also reported that the *sarX* gene in *S. aureus* expressed maximally and temporally at the transcriptional level in stationary phase [60]. The *agr* transcripts, RNAII and RNAIII, has been found to have significantly increased expression in a *sarX* mutant. SarX has been discovered to negatively control *agr* expression by binding to the *agr* promoter, which in turn displays strain-specific effects on regulating the biofilm formation. This finding seems to contradict the simultaneously increased expression of AgrC and SarX in CA-SA ST398 group. The transcription factor sigma B (SigB) is reported to influence the expressional production of several genes encoding stress-response proteins and virulence factors, and appears to counteract the *agr* system on its effect on the expressional production of virulent factors [61]. It also contradicts the simutaneously elevated expression of AgrC and SigB in CA-SA ST398 group. As for the SrrB and YycG, the AgrA/C, SrrA/B, YycG/H systems are independently well-known two-component regulatory systems. There are also no report showing the interaction between AgrC and SrrB or YycG. However, in view of the active interaction sources of STRING database include Co-occurrence, Gene Fusion, Neighborhood, Co-expression, Databases, Experiments and Textmining, it would be possible for us to fail to find the related experiments to support our results. In this model, AgrC seems to be the central nodes in CA-SA ST398 up-regulated network, indicating the contribution of Agr to the high virulence in CA-SA ST398. Unfortunately, we failed to confirm the higher expression of AgrC in CA-SA ST398 group because of the lack of AgrC antibody.

Notably, the higher amounts of *agrC* transcripts are present in CA-SA ST398 isolates detected by the LC MS/MS and identified by RT-PCR, albeit of the fact that *agrC* and *agrA* are both part of the same polycistronic transcript, RNAII. The transcriptional difference might come from the experimental noise or the prompt response of AgrC to autoinducing peptide (AIP, the AgrC ligand) in CA-SA ST398 lineage than other lineages. As for the discrepancy between the transcriptional and translational level of *agrC*/*agrA* expression ratio. We have checked that both of primer pairs for *agrA* and *agrC* have optimal primer efficiencies (> 90%) in their qRT-PCR setup, although the primer efficiency of *agrC* (~ 97%) is higher than that of *agrA* (~ 92%). The factor of primer efficiency might contribute little to the expression ratio discrepancy between the transcriptional and translational level. Therefore, the above reasons of post modification and significant noise could be more prone to lead to this difference.

Additionally, there could be two possible reasons for the other RNAII encoded factors AgrB and AgrD missing in the up-regulated in ST398 isolates. (1) Because of the sensitivity of the method, AgrD (~ 5KD) has low molecular weight to be detected by the LC-MS/MS method. (2) The small peptide of AIP produced by AgrD was secreted to the supernatant. (3) There could be one or several mutations in the *agrB* gene causing the change of amino acid in AgrB (~ 26 kDa) and the mutated AgrB protein couldn’t match to the template (S0385 and TW20).

Moreover, SigB should be co-transcribed with the anti-sigma factors (RsbV and RsbW) [62]. We have detected the RsbV and RsbW proteins by the LC-MS/MS in our original data. However, our Table 3 shows the differentially expressed proteins with a P value by Student’s t-test lower than 0.05 and with an ST239/ST398 change higher than 1.5-fold or lower than 0.67-fold. Due to our strict screening rule, although RsbV and RsbW were up-regulated in some isolates of CA-SA ST398, their expressions are not significant according to P value by Student’s t-test.

Interestingly, the highest fold-change in expression of penicillin-binding protein 3 (Pbp3) between HA-SA ST239 and CA-SA ST398 isolates may account for the inferior fitness of ST239. Compared to the four HA-MRSA ST239 isolates, only half of the four CA-SA ST398 isolates are MRSA. However, instead of Pbp2a, Pbp3 was highly expressed in HA-MRSA ST239 group. One possible illustration for the striking Pbp3 expression might be due to the polymorphism of *pbp3* in HA-MRSA ST239. Chadwick et al has reported that two nucleotide polymorphisms (G88A and G2047A) in the *pbp3* gene of *S. aureus* were highly associated with the CA-MRSA USA300 lineage [63]. The nucleotide polymorphism need to be verified by gene sequencing. Another possibility may be due to fluoroquinolone connection in HA-MRSA ST239. It is well-established that HA-MRSA strains are more resistant to various groups of antibiotics such as fluoroquinolones than CA-MRSA. Diverse fitness cost associated with high-level resistance to fluoroquinolones was demonstrated by multiple groups to contribute to the clonal dynamics of HA-MRSA [64–66]. The genetic basis of differing vitality has also been partly established [64, 67]. The connection of the fitness cost of the HA-MRSA ST239 lineage and fluoroquinolone resistance needs to be further studied.

Technically, our study was based on quantitative proteome analysis of multiple pathogenic polypeptides of four HA-SA ST239 and four CA-SA ST398 isolates simultaneously, which is a good supplement to the recently published proteomic analysis [68]. One theoretical boundedness is isolate-dependence, which might demand the recharacterization of each single strain. Nevertheless, the identical concept is effective for any phylogenic or physiologic characterization of any isolate.

 Taken together, biologically, we could conclude that the higher expression of Agr system and its interactive factors (PhoP, SrrB, YycG, SarX, SigB and ClpP) based on the protein-protein interaction network in the CA-SA ST398 strains may improve the acute infection of CA-SA, while the higher expression of *agr*-negative regulating surface-related factors (FnbpA, SpA, Atl, ClfA, IsaA, IsaB, LtaS, SsaA and Cna) contribute to the durative infection of HA-SA.

## Additional files

**Additional file 1.** The detailed information on differentially expressed 209 proteins in each isolate.

**Additional file 2.** Potential interactions based on 64 up-regulated genes in the HA-SA ST239 group and 145 up-regulated genes in the CA-SA ST398 group using the STRING (version 10.0) database.
