# Supplementary material for: Distinct virulent network between healthcare- and community-associated Staphylococcus aureus based on proteomic analysis
Source: Clin Proteomics. 2018 Jan 8;15:2. doi: 10.1186/s12014-017-9178-5 (PMC5757299; doi:10.1186/s12014-017-9178-5)
Supplement: Supplementary file 1 — Additional file 1. The detailed information on differentially expressed 209 proteins in each isolate. [file 12014_2017_9178_MOESM1_ESM.docx]

**Additional file 1**

**Table S1.** Up-regulated proteins in ST239 group compared to ST398 group

| **Primer name** | **Sequence (5' to 3')** |
| --- | --- |
| *spa-F* | ACGGCACTACTGCTGACAAA |
| *spa-R* | GCATGGTTTGCTGGTTGCTT |
| *isaA-F* | CTGCAGGTGCTACTGGTTCA |
| *isaA-R* | TGAAGCACCTGATGGGTTGT |
| *isaB-F* | GTGCAGCAACGACATTAGCA |
| *isaB-R* | CCGCCTGTGCTTCTTGATGT |
| *cna-F* | AACAGATGCAAACGGTATTGCTAA |
| *cna-R* | GTGCTGGCGCCTCTATTTCT |
| *fnbpA-F* | ACAACTGCAACTAACGTTAATCAT |
| *fnbpA-R* | TGCTGGTTGTGCAGTTTGTG |
| *atl-F* | AAAGCAATCGTTGGTGGTGC |
| *atl-R* | TGGTGTGTTCCTGGATGTGC |
| *sigB-F* | ATGGGGCAACAAGATGACCA |
| *sigB-R* | CCGATACGCTCACCTGTCTC |
| *srrB-F* | TCGCTTGCCATTGTCCTTGA |
| *srrB-R* | CGGATAACCCTTCAGCATCCA |
| *gyrB-F* | CAAATGATCACAGCATTTGGTACAG |
| *gyrB-R* | CGGCATCAGTCATAATGACGAT |
| *ssaA-F* | CGGTTCAACTTGGGGCAATG |
| *ssaA-R* | AACGTGACCGTATGCACCTT |
| *clfA-F* | CCAGAAAACTTTGAGGATGTCACTA |
| *clfA-R* | TGCTATTCGGATCAATATGACCA |
| *sarX-F* | TGAGAAATTAGAAACATTGCTTGGC |
| *sarX-R* | TCTAGCTCATCCATTGCAGTT |
| *agrA-F* | GGAAATTGCCCTCGCAACTG |
| *agrA-R* | CCAACTGGGTCATGCTTACG |
| *agrC-F* | CGAAATGCGCAAGTTCCGTC |
| *agrC-R* | AGGCCAGGCATGTCATCTTC |
| *clpP-F* | GGTATGGCTGCATCAATGGG |
| *clpP-R* | CTTGTCCTTGAGCACCACCT |
| *yycG-F* | AGCGTCGTGAATTTGTTGCC |
| *yycG-R* | TGGCGCAAGTTCCTCATCTT |
| *ltaS-F* | AGCGTCGTGAATTTGTTGCC |
| *ltaS-R* | TGGCGCAAGTTCCTCATCTT |
| *phoP-F* | AGCGTCGTGAATTTGTTGCC |
| *phoP-R* | TGGCGCAAGTTCCTCATCTT |

**Table S2.** Up-regulated proteins in ST239 group compared to ST398 group

| **Uniport acc no.** | **Entrez GeneID** | **Protein description** | **Protein name** | **prot_mass** | **ST239-1** | **ST239-2** | **ST239-3** | **ST239-4** | **ST398-1** | **ST398-2** | **ST398-3** | **ST398-4** | **FC 239/398** | **P-value** |
| --- | --- | --- | --- | --- | --- | --- | --- | --- | --- | --- | --- | --- | --- | --- |
| **Known virulence-related factors** | | | | | | | | | | | | | | |
| D2NA85 | 12321479 | Fibronectin-binding protein A | FnbpA | 109066 | 14 | 29 | 3 | 7 | 2 | 1 | 1 | 1 | 13.99 | 0.018 |
| D2N8V1 | 12322087 | Aerolysin/Leukocidin family protein | Aerolysin/Leukocidin family protein | 40348 | 22 | 46 | 25 | 88 | 4 | 10 | 4 | 8 | 7.10 | 0.02 |
| D2N8V0 | 12322086 | Synergohymenotropic toxin | Synergohymenotropic toxin | 38580 | 6 | 18 | 17 | 22 | 4 | 2 | 1 | 5 | 5.30 | 0.021 |
| D2N3G2 | 12323114 | ImmunoGlobulin g binding protein a | SpA | 52961 | 104 | 275 | 111 | 325 | 1 | 195 | 54 | 1 | 3.26 | 0.032 |
| D2NAS2 | 12321666 | Collagen adhesin | Cna | 133085 | 26 | 66 | 40 | 44 | 16 | 23 | 21 | 10 | 2.54 | 0.021 |
| D2N627 | 12322372 | Bifunctional autolysin | Atl | 136681 | 604 | 877 | 518 | 381 | 230 | 321 | 236 | 181 | 2.46 | 0.021 |
| D2N5J4 | 12321080 | Clumping factor A | ClfA | 64504 | 198 | 230 | 99 | 89 | 86 | 72 | 87 | 46 | 2.11 | 0.021 |
| **Staphylococcal antigen** | | | | | | | | | | | | | | |
| D2N9N2 | 12321276 | Staphylococcal secretory antigen ssaA | SsaA | 29577 | 141 | 286 | 73 | 241 | 31 | 55 | 42 | 30 | 4.70 | 0.021 |
| D2NAF1 | 12321545 | Immunodominant staphylococcal antigen A | IsaA | 24207 | 82 | 148 | 47 | 69 | 31 | 37 | 53 | 18 | 2.50 | 0.043 |
| D2NAM0 | 12321614 | Immunodominant staphylococcal antigen B | IsaB | 19509 | 31 | 47 | 24 | 30 | 17 | 23 | 20 | 16 | 1.73 | 0.021 |
| **Other known proteins** | | | | | | | | | | | | | | |
| D2N382 | 12323034 | Penicillin-binding protein 3 (Pbp 3) (Pspb20) | Pbp 3 | 76171 | 170 | 168 | 287 | 102 | 1 | 9 | 1 | 1 | 69.88 | 0.018 |
| D2N4Y0 | 12320865 | Acetyl-CoA acetyltransferase | Acetyl-CoA acetyltransferase | 41030 | 26 | 26 | 17 | 12 | 2 | 1 | 1 | 3 | 12.51 | 0.019 |
| D2NAM6 | 12321620 | N-acetylmuramoyl-L-alanine amidase | N-acetylmuramoyl-L-alanine amidase | 69170 | 63 | 100 | 67 | 83 | 5 | 18 | 7 | 1 | 10.41 | 0.021 |
| D2N4M0 | 12320745 | N-acetylmuramoyl-L-alanine amidase Sle1 | N-acetylmuramoyl-L-alanine amidase Sle1 | 35993 | 33 | 94 | 17 | 117 | 2 | 16 | 9 | 1 | 9.41 | 0.021 |
| D2N5C6 | 12321011 | Anion-binding protein | LtaS | 74399 | 31 | 52 | 12 | 22 | 6 | 14 | 8 | 2 | 4.03 | 0.043 |
| D2N9E7 | 12322298 | HAD-superfamily hydrolase, subfamily IIB | HAD-superfamily hydrolase, subfamily IIB | 32101 | 21 | 9 | 16 | 9 | 7 | 3 | 3 | 2 | 3.87 | 0.019 |
| D2N751 | 12322747 | Peptide methionine sulfoxide reductase MsrA | MsrA | 20588 | 8 | 11 | 38 | 13 | 5 | 5 | 5 | 4 | 3.75 | 0.018 |
| D2NAT9 | 12321683 | tRNA modification GTPase MnmE | MnmE | 51341 | 29 | 23 | 24 | 16 | 8 | 4 | 8 | 5 | 3.74 | 0.02 |
| D2N835 | 12321785 | Universal stress protein family, putative | Universal stress protein family, putative | 15226 | 184 | 121 | 278 | 139 | 38 | 58 | 53 | 49 | 3.66 | 0.021 |
| D2N6J2 | 12322538 | Ribosomal RNA small subunit methyltransferase B | Sun | 49975 | 11 | 10 | 4 | 11 | 2 | 6 | 1 | 2 | 3.51 | 0.041 |
| D2N9E2 | 12322293 | Morphine 6-dehydrogenase (Naloxone reductase) | Morphine 6-dehydrogenase (Naloxone reductase) | 32215 | 37 | 33 | 15 | 16 | 13 | 5 | 8 | 4 | 3.46 | 0.021 |
| D2NAH7 | 12321571 | Aspartate 1-decarboxylase | PanD | 14073 | 4 | 2 | 3 | 1 | 1 | 1 | 1 | 1 | 3.05 | 0.047 |
| D2N655 | 12322400 | Protein YwbD | YwbD | 44912 | 14 | 8 | 16 | 15 | 4 | 5 | 3 | 6 | 2.95 | 0.021 |
| D2NAK8 | 12321602 | Alkaline phosphatase 3 (Alkaline phosphataseIII) (APase III) | Alkaline phosphatase 3 (Alkaline phosphataseIII) (APase III) | 51986 | 2 | 2 | 1 | 3 | 1 | 1 | 1 | 1 | 2.80 | 0.046 |
| D2NAM5 | 12321619 | Phage infection protein | Phage infection protein | 108777 | 47 | 69 | 30 | 33 | 18 | 16 | 20 | 11 | 2.77 | 0.021 |
| D2N7Y0 | 12321730 | Holliday junction ATP-dependent DNA helicase RuvB | RuvB | 37688 | 5 | 7 | 4 | 4 | 2 | 1 | 1 | 4 | 2.75 | 0.037 |
| D2N917 | 12322156 | Cardiolipin synthetase (Cardiolipin synthase) (CLsynthase) | Cardiolipin synthetase (Cardiolipin synthase) (CLsynthase) | 56503 | 17 | 7 | 10 | 7 | 4 | 4 | 4 | 4 | 2.58 | 0.013 |
| D2N7V5 | 12323001 | UPF0473 protein | UPF0473 protein | 11949 | 11 | 24 | 10 | 14 | 6 | 8 | 3 | 6 | 2.58 | 0.02 |
| D2N410 | 12323312 | 5~-nucleotidase, lipoprotein e(P4) family | 5~-nucleotidase, lipoprotein e (P4) family | 33352 | 19 | 47 | 14 | 37 | 13 | 15 | 9 | 10 | 2.47 | 0.043 |
| D2N9P4 | 12321288 | Inositol monophosphatase family protein | Inositol monophosphatase family protein | 30416 | 12 | 18 | 12 | 13 | 5 | 7 | 7 | 4 | 2.40 | 0.019 |
| D2N6X6 | 12322672 | Glycine betaine transporter OpuD | Glycine betaine transporter OpuD | 60471 | 8 | 8 | 8 | 6 | 1 | 6 | 4 | 3 | 2.31 | 0.026 |
| D2N6I0 | 12322526 | PhnB protein | PhnB | 14873 | 17 | 11 | 11 | 10 | 8 | 6 | 6 | 3 | 2.24 | 0.019 |
| D2N953 | 12322192 | Putative transcriptional regulator superfamily | Putative transcriptional regulator superfamily | 12686 | 11 | 7 | 8 | 9 | 6 | 5 | 4 | 2 | 2.10 | 0.021 |
| D2N755 | 12322751 | Protein YphP | YphP | 16070 | 14 | 13 | 8 | 10 | 9 | 5 | 6 | 1 | 2.10 | 0.043 |
| D2N5A8 | 12320993 | N-acetylglucosamine-6-phosphate deacetylase | NagA | 43205 | 8 | 5 | 7 | 7 | 2 | 2 | 5 | 4 | 2.10 | 0.027 |
| D2N7I6 | 12322882 | Segregation and condensation protein B | ScpB | 20142 | 5 | 7 | 4 | 4 | 4 | 3 | 1 | 3 | 1.97 | 0.037 |
| D2N750 | 12322746 | Peptide methionine sulfoxide reductase MsrB | MsrB | 16263 | 11 | 15 | 38 | 14 | 11 | 11 | 11 | 6 | 1.94 | 0.046 |
| D2N657 | 12322402 | Phosphocarrier protein HPr (Histidine-containing protein) | Phosphocarrier protein HPr (Histidine-containing protein) | 9496 | 111 | 135 | 161 | 108 | 56 | 78 | 78 | 56 | 1.92 | 0.019 |
| D2N6I4 | 12322530 | DNA-directed RNA polymerase subunit omega | RpoZ | 8150 | 18 | 24 | 11 | 15 | 9 | 9 | 13 | 5 | 1.84 | 0.042 |
| D2N9L8 | 12321262 | Butyryl-CoA dehydrogenase | Butyryl-CoA dehydrogenase | 42948 | 18 | 18 | 23 | 36 | 14 | 14 | 13 | 11 | 1.84 | 0.019 |
| D2N6L2 | 12322558 | UPF0122 protein | UPF0122 protein | 13581 | 6 | 9 | 5 | 5 | 4 | 4 | 5 | 2 | 1.81 | 0.037 |
| D2N7K7 | 12322903 | Lipoamide acyltransferase component of branched-chain alpha-keto aciddehydrogenase complex (Dihydrolipoyllysine-residue (2-methylpropanoyl)transferase) (E2) (Dihydrolipoamide branched chaintransacylase) OS | Lipoamide acyltransferase component of branched-chain alpha-keto aciddehydrogenase complex | 46770 | 14 | 11 | 11 | 12 | 4 | 10 | 7 | 6 | 1.80 | 0.02 |
| D2N6J3 | 12322539 | Probable dual-specificity RNA methyltransferase RlmN | RlmN | 41904 | 12 | 11 | 10 | 7 | 7 | 5 | 5 | 6 | 1.80 | 0.028 |
| D2N681 | 12322426 | Inositol-1-monophosphatase (IMPase) (Inositol-1-phosphatase) (I-1-Pase) | IMPase | 30476 | 12 | 18 | 12 | 13 | 7 | 7 | 9 | 8 | 1.76 | 0.019 |
| D2N5S9 | 12321165 | ATP-dependent helicase/nuclease subunit A | AddA | 141267 | 30 | 24 | 24 | 22 | 19 | 16 | 14 | 9 | 1.73 | 0.02 |
| D2N5P1 | 12321127 | HAD-superfamily subfamily IIA hydrolase | HAD-superfamily subfamily IIA hydrolase | 27979 | 31 | 23 | 39 | 28 | 23 | 11 | 19 | 18 | 1.69 | 0.029 |
| D2N7P4 | 12322940 | Superoxide dismutase | Superoxide dismutase | 22711 | 129 | 121 | 173 | 137 | 97 | 95 | 80 | 62 | 1.68 | 0.021 |
| D2N7T3 | 12322979 | Ribosomal silencing factor RsfS | RsfS | 13452 | 14 | 18 | 16 | 15 | 13 | 7 | 14 | 4 | 1.66 | 0.029 |
| D2N842 | 12321792 | Probable thiol peroxidase | Tpx | 18004 | 54 | 53 | 51 | 54 | 33 | 40 | 23 | 34 | 1.64 | 0.02 |
| D2N7J2 | 12322888 | Oxidoreductase | Oxidoreductase | 33514 | 18 | 14 | 14 | 12 | 9 | 10 | 9 | 8 | 1.60 | 0.019 |
| D2N941 | 12322180 | UDP-N-acetylglucosamine 2-epimerase | UDP-N-acetylglucosamine 2-epimerase | 42404 | 6 | 6 | 6 | 7 | 4 | 3 | 5 | 4 | 1.60 | 0.017 |
| D2N7L6 | 12322912 | N utilization substance protein B homolog | NusB | 15061 | 13 | 13 | 12 | 11 | 7 | 9 | 9 | 6 | 1.58 | 0.019 |
| D2N6K8 | 12322554 | Acyl carrier protein | AcpP | 8549 | 27 | 32 | 25 | 28 | 20 | 24 | 12 | 15 | 1.58 | 0.021 |
| D2N4I9 | 12320714 | Type I restriction-modification system, M subunit | HsdM | 59452 | 37 | 29 | 29 | 30 | 17 | 15 | 24 | 23 | 1.58 | 0.02 |
| D2N4U4 | 12320829 | 50S ribosomal protein L10 | RplJ | 17710 | 85 | 105 | 93 | 106 | 68 | 76 | 47 | 58 | 1.55 | 0.021 |
| D2N6F6 | 12322502 | UDP-N-acetylmuramoylalanine--D-glutamate ligase | MurD | 49843 | 42 | 42 | 36 | 36 | 29 | 20 | 28 | 24 | 1.54 | 0.019 |
| D2N5D9 | 12321024 | Ribonucleoside-diphosphate reductase, beta subunit | Ribonucleoside-diphosphate reductase, beta subunit | 37513 | 98 | 95 | 89 | 87 | 56 | 50 | 73 | 61 | 1.54 | 0.021 |
| D2N8L2 | 12321998 | Aminopeptidase PepS | Aminopeptidase PepS | 46919 | 79 | 44 | 67 | 64 | 39 | 39 | 44 | 43 | 1.54 | 0.028 |
| **Unknown proteins** | | | | | | | | | | | | | | |
| D2N9M8 | 12321272 | Uncharacterized protein | Uncharacterized protein | 12577 | 32 | 44 | 31 | 28 | 11 | 19 | 1 | 6 | 3.63 | 0.021 |
| D2N5P0 | 12321126 | Uncharacterized protein | Uncharacterized protein | 16823 | 17 | 14 | 12 | 7 | 6 | 8 | 5 | 2 | 2.49 | 0.043 |
| D2N4W0 | 12320845 | Uncharacterized protein | Uncharacterized protein | 24943 | 19 | 17 | 12 | 10 | 6 | 9 | 8 | 4 | 2.17 | 0.021 |
| D2NA92 | 12321486 | Uncharacterized protein | Uncharacterized protein | 16034 | 78 | 63 | 73 | 53 | 49 | 26 | 32 | 38 | 1.85 | 0.021 |
| D2N685 | 12322430 | Uncharacterized protein | Uncharacterized protein | 18581 | 16 | 11 | 16 | 9 | 8 | 7 | 10 | 7 | 1.62 | 0.041 |
| D2N588 | 12320973 | Uncharacterized protein | Uncharacterized protein | 20889 | 19 | 21 | 26 | 16 | 12 | 13 | 10 | 15 | 1.61 | 0.021 |

**Table S3.** Up-regulated proteins in ST398 group compared to ST239 group

| **Uniport acc no.** | **Entrez GeneID** | **Protein description** | **Protein name** | **prot_mass** | **ST239-1** | **ST239-2** | **ST239-3** | **ST239-4** | **ST398-1** | **ST398-2** | **ST398-3** | **ST398-4** | **FC 398/239** | **P-value** |
| --- | --- | --- | --- | --- | --- | --- | --- | --- | --- | --- | --- | --- | --- | --- |
| **Known virulence-related factors** | | | | | | | | | | | | | | |
| D2N8W5 | 12322101 | AgrC | AgrC | 25702 | 1 | 1 | 1 | 1 | 18 | 14 | 16 | 13 | 23.913 | 0.014 |
| D2N8W6 | 12322102 | Accessory gene regulator protein A | AgrA | 27906 | 1 | 1 | 1 | 11 | 23 | 22 | 26 | 25 | 7.200 | 0.018 |
| D2N7I3 | 12322879 | Sensor protein SrrB (Staphylococcal respiratory responseprotein B) | SrrB | 63881 | 11 | 9 | 6 | 5 | 13 | 14 | 15 | 21 | 2.024 | 0.021 |
| D2N8Z3 | 12322134 | RNA polymerase sigma factor | sigB | 29429 | 4 | 1 | 2 | 2 | 4 | 5 | 5 | 5 | 1.983 | 0.025 |
| D2N718 | 12322714 | Conserved virulence factor B (SAPIG1392) | Conserved virulence factor B | 34184 | 3 | 3 | 8 | 11 | 12 | 12 | 12 | 13 | 1.976 | 0.017 |
| D2N5H5 | 12321061 | ATP-dependent Clp protease proteolytic subunit | clpP | 21513 | 21 | 15 | 21 | 18 | 28 | 49 | 26 | 31 | 1.768 | 0.02 |
| **Transcriptional regulator** | | | | | | | | | | | | | | |
| D2N576 | 12320961 | HTH-type transcriptional regulator SarX (Staphylococcal accessoryregulator X) | SarX | 14205 | 1 | 1 | 1 | 1 | 6 | 9 | 10 | 5 | 9.910 | 0.014 |
| D2N9W6 | 12321360 | AraC family regulatory protein | AraC family regulatory protein | 81888 | 1 | 1 | 1 | 1 | 6 | 2 | 4 | 3 | 4.481 | 0.014 |
| D2N9P8 | 12321292 | Phosphosugar-binding transcriptional regulator, RpiR family | Phosphosugar-binding transcriptional regulator, RpiR family | 33020 | 2 | 2 | 3 | 4 | 9 | 14 | 16 | 7 | 3.993 | 0.02 |
| D2N3I9 | 12323141 | Transcriptional regulator, GntR family | Transcriptional regulator, GntR family | 28541 | 1 | 1 | 1 | 2 | 6 | 3 | 5 | 3 | 3.321 | 0.017 |
| D2N9Q7 | 12321301 | Phosphosugar-binding transcriptional regulator | Phosphosugar-binding transcriptional regulator | 29794 | 1 | 1 | 1 | 3 | 4 | 3 | 7 | 6 | 3.074 | 0.026 |
| D2N823 | 12321773 | Alkaline phosphatase synthesis transcriptional regulatory protein phoP | PhoP | 27021 | 3 | 2 | 4 | 5 | 9 | 9 | 9 | 8 | 2.362 | 0.018 |
| D2N6S6 | 12322622 | Glycerol uptake operon antiterminator regulatory protein | Glycerol uptake operon antiterminator regulatory protein | 20108 | 2 | 1 | 4 | 3 | 6 | 8 | 4 | 6 | 2.410 | 0.028 |
| D2N8K6 | 12321992 | Regulatory protein RecX | RecX | 32245 | 3 | 1 | 1 | 2 | 3 | 3 | 5 | 4 | 2.077 | 0.037 |
| **Arginine and proline metabolism** | | | | | | | | | | | | | | |
| D2NAL7 | 12321611 | Arginine deiminase | ArcA | 46928 | 1 | 5 | 7 | 1 | 7 | 17 | 23 | 34 | 6.112 | 0.028 |
| D2NAL6 | 12321610 | Ornithine carbamoyltransferase, catabolic | ArcB | 37762 | 4 | 1 | 11 | 1 | 15 | 17 | 20 | 19 | 4.184 | 0.02 |
| D2NAL4 | 12321608 | Carbamate kinase | ArcC | 34381 | 1 | 1 | 5 | 2 | 5 | 6 | 7 | 6 | 2.525 | 0.027 |
| **Multidrug resistance** | | | | | | | | | | | | | | |
| D2N9T7 | 12321331 | Multidrug resistance protein A | Multidrug resistance protein A | 23026 | 1 | 1 | 2 | 3 | 8 | 8 | 7 | 7 | 4.725 | 0.019 |
| D2N552 | 12320937 | Multidrug resistance ABC transporter ATP-binding and permease protein | Multidrug resistance ABC transporter ATP-binding and permease protein | 64050 | 3 | 5 | 3 | 13 | 23 | 21 | 24 | 23 | 3.844 | 0.019 |
| **Two-component system** | | | | | | | | | | | | | | |
| D2N9Y1 | 12321375 | Nitrate reductase, beta subunit | NarH | 55305 | 4 | 5 | 1 | 1 | 6 | 9 | 6 | 5 | 2.357 | 0.027 |
| D2N361 | 12323013 | Sensor protein YycG | YycG | 69923 | 11 | 10 | 7 | 5 | 17 | 11 | 14 | 21 | 1.888 | 0.029 |
| **Pyrimidine metabolism** | | | | | | | | | | | | | | |
| D2NAJ6 | 12321590 | Anaerobic ribonucleoside-triphosphate reductase | NrdD | 70509 | 3 | 1 | 1 | 1 | 20 | 8 | 22 | 13 | 11.836 | 0.018 |
| D2N6H8 | 12322524 | Orotate phosphoribosyltransferase | PyrE | 22057 | 2 | 2 | 8 | 3 | 10 | 9 | 9 | 9 | 2.356 | 0.017 |
| D2N6H3 | 12322519 | Aspartate carbamoyltransferase | PyrB | 33257 | 2 | 3 | 5 | 3 | 6 | 4 | 9 | 10 | 2.107 | 0.042 |
| D2N942 | 12322181 | Uracil phosphoribosyltransferase | Upp | 23050 | 56 | 66 | 66 | 73 | 112 | 86 | 102 | 100 | 1.524 | 0.02 |
| **Pyruvate metabolism** | | | | | | | | | | | | | | |
| D2N3S5 | 12323227 | Formate acetyltransferase | PflB | 84848 | 177 | 215 | 408 | 129 | 529 | 491 | 267 | 910 | 2.367 | 0.043 |
| D2N9V0 | 12321344 | Probable malate:quinone oxidoreductase | Mqo | 54870 | 17 | 10 | 12 | 4 | 29 | 24 | 28 | 18 | 2.261 | 0.021 |
| D2N7L8 | 12322914 | Acetyl-CoA carboxylase, biotin carboxylase | AccC | 50063 | 8 | 6 | 9 | 11 | 13 | 12 | 18 | 17 | 1.777 | 0.021 |
| **Histidine metabolism** | | | | | | | | | | | | | | |
| D2N9R5 | 12321309 | Urocanate hydratase | HutU | 60626 | 1 | 1 | 1 | 1 | 11 | 13 | 11 | 8 | 17.435 | 0.013 |
| D2N9R4 | 12321308 | Imidazolonepropionase | HutI | 45080 | 1 | 1 | 2 | 2 | 7 | 6 | 7 | 4 | 3.592 | 0.019 |
| D2N9R7 | 12321311 | Formimidoylglutamase | HutG | 34450 | 11 | 11 | 17 | 6 | 30 | 14 | 21 | 18 | 1.821 | 0.042 |
| **Glycolysis/Gluconeogensis** | | | | | | | | | | | | | | |
| D2NAA0 | 12321494 | Fructose-1,6-bisphosphatase class 3 | Fbp | 76213 | 12 | 16 | 23 | 3 | 31 | 41 | 32 | 38 | 2.628 | 0.021 |
| D2N9Z9 | 12321393 | 2,3-bisphosphoglycerate-dependent phosphoglycerate mutase | GpmA | 26680 | 56 | 52 | 59 | 48 | 160 | 94 | 128 | 155 | 2.505 | 0.021 |
| D2N8B5 | 12321865 | Phosphoenolpyruvate carboxykinase [ATP] | PckA | 59392 | 49 | 35 | 49 | 12 | 82 | 99 | 78 | 81 | 2.342 | 0.02 |
| **Other known proteins** | | | | | | | | | | | | | | |
| D2N3K5 | 12323157 | Putative tyrosine-protein phosphatase CapC | CapC | 29362 | 1 | 1 | 1 | 1 | 22 | 7 | 8 | 20 | 21.851 | 0.014 |
| D2N3F9 | 12323111 | Antigen, 67 kDa | Antigen, 67 kDa | 67593 | 1 | 1 | 1 | 1 | 14 | 4 | 1 | 18 | 11.844 | 0.047 |
| D2N5J2 | 12321078 | Probable membrane protein | Probable membrane protein | 19946 | 1 | 1 | 1 | 1 | 8 | 8 | 4 | 2 | 8.221 | 0.013 |
| D2NA54 | 12321448 | 3-oxoacyl-[acyl-carrier-protein] reductase (3-ketoacyl-acyl carrier protein reductase) | 3-oxoacyl-[acyl-carrier-protein] reductase | 29445 | 1 | 1 | 1 | 1 | 7 | 5 | 6 | 3 | 7.785 | 0.014 |
| D2N5B9 | 12321004 | 7-cyano-7-deazaguanine synthase | QueC | 24887 | 1 | 1 | 1 | 1 | 7 | 5 | 7 | 6 | 6.591 | 0.013 |
| D2N3D6 | 12323088 | Amidohydrolase 2 | Amidohydrolase 2 | 36923 | 3 | 6 | 3 | 9 | 17 | 23 | 16 | 77 | 6.507 | 0.02 |
| D2N543 | 12320928 | Iron dependent repressor | Iron dependent repressor | 24884 | 1 | 1 | 3 | 1 | 8 | 6 | 13 | 9 | 6.417 | 0.018 |
| D2N8W0 | 12322096 | Nitroreductase family protein | Nitroreductase family protein | 24004 | 3 | 1 | 4 | 6 | 20 | 22 | 28 | 17 | 6.124 | 0.021 |
| D2N628 | 12322373 | Acetyl transferase | Acetyl transferase | 16493 | 1 | 1 | 2 | 1 | 5 | 7 | 5 | 4 | 5.957 | 0.017 |
| D2NA36 | 12321430 | Para-nitrobenzyl esterase | Para-nitrobenzyl esterase | 51980 | 4 | 1 | 4 | 2 | 14 | 12 | 13 | 22 | 5.364 | 0.02 |
| D2N4R9 | 12320795 | Dihydropteroate synthase | FolP | 29477 | 2 | 2 | 6 | 9 | 14 | 18 | 36 | 23 | 4.827 | 0.02 |
| D2N3T8 | 12323240 | ABC transporter, substrate-binding protein | ABC transporter, substrate-binding protein | 55481 | 4 | 1 | 1 | 2 | 11 | 6 | 6 | 13 | 4.823 | 0.019 |
| D2N549 | 12320934 | Teichoic acid biosynthesis protein X | Teichoic acid biosynthesis protein X | 32332 | 1 | 1 | 1 | 1 | 2 | 4 | 6 | 4 | 4.808 | 0.013 |
| D2N944 | 12322183 | UPF0340 protein | UPF0340 protein | 18879 | 1 | 1 | 1 | 5 | 8 | 9 | 9 | 6 | 4.721 | 0.017 |
| D2N4D0 | 12320655 | Stage 0 sporulation protein J | Stage 0 sporulation protein J | 32468 | 1 | 1 | 1 | 1 | 4 | 3 | 3 | 2 | 4.545 | 0.013 |
| D2N5Q4 | 12321140 | Cytosol aminopeptidase family protein | Cytosol aminopeptidase family protein | 54173 | 1 | 1 | 1 | 23 | 25 | 41 | 28 | 22 | 4.529 | 0.038 |
| D2N4P0 | 12320769 | Stage 0 sporulation protein YaaT | Stage 0 sporulation protein YaaT | 30219 | 3 | 1 | 1 | 1 | 8 | 5 | 7 | 6 | 3.979 | 0.018 |
| D2N526 | 12320911 | Hydrolase, alpha/beta hydrolase fold family | Hydrolase, alpha/beta hydrolase fold family | 30172 | 1 | 1 | 1 | 3 | 5 | 8 | 7 | 4 | 3.915 | 0.018 |
| D2N723 | 12322719 | 4-hydroxy-tetrahydrodipicolinate reductase | DapB | 26658 | 1 | 1 | 3 | 2 | 8 | 4 | 4 | 12 | 3.803 | 0.019 |
| D2N3Q0 | 12323202 | Pts system eiibc component | Pts system eiibc component | 50672 | 1 | 3 | 2 | 2 | 8 | 6 | 6 | 11 | 3.711 | 0.019 |
| D2N8G9 | 12321927 | ABC transporter EcsB | ABC transporter EcsB | 48502 | 1 | 1 | 1 | 1 | 4 | 2 | 2 | 4 | 3.671 | 0.013 |
| D2N3F6 | 12323108 | Aminoacylase | Aminoacylase | 43019 | 10 | 6 | 8 | 10 | 18 | 15 | 13 | 71 | 3.580 | 0.02 |
| D2N585 | 12320970 | Lipoprotein, putative | Lipoprotein, putative | 14699 | 2 | 1 | 1 | 2 | 7 | 6 | 5 | 3 | 3.474 | 0.019 |
| D2N6B0 | 12322455 | SpoU rRNA Methylase family protein | SpoU rRNA Methylase family protein | 26974 | 3 | 1 | 4 | 5 | 13 | 6 | 11 | 16 | 3.420 | 0.021 |
| D2N349 | 12321704 | ADP-dependent (S)-NAD(P)H-hydrate dehydratase | NnrD | 29549 | 1 | 1 | 2 | 2 | 4 | 4 | 5 | 9 | 3.388 | 0.019 |
| D2NAA6 | 12321500 | Glyoxalase family protein | Glyoxalase family protein | 30083 | 4 | 2 | 2 | 2 | 7 | 9 | 12 | 8 | 3.372 | 0.018 |
| D2N362 | 12323014 | YycH protein | YycH protein | 50921 | 1 | 1 | 2 | 1 | 2 | 5 | 4 | 6 | 3.211 | 0.026 |
| D2N6F0 | 12322496 | UPF0747 protein | UPF0747 protein | 62880 | 2 | 1 | 1 | 3 | 6 | 4 | 7 | 5 | 3.177 | 0.02 |
| D2N3K2 | 12323154 | Aldehyde-alcohol dehydrogenase 2 | Aldehyde-alcohol dehydrogenase 2 | 94972 | 53 | 58 | 45 | 93 | 199 | 150 | 125 | 302 | 3.118 | 0.021 |
| D2N9Q3 | 12321297 | Phosphoglycolate phosphatase | Phosphoglycolate phosphatase | 23586 | 3 | 1 | 1 | 2 | 5 | 6 | 7 | 5 | 3.045 | 0.019 |
| D2N411 | 12323313 | ABC transporter, permease protein | ABC transporter, permease protein | 38449 | 4 | 2 | 1 | 4 | 13 | 6 | 9 | 5 | 3.012 | 0.02 |
| D2N7J1 | 12322887 | Adp-ribose pyrophosphatase (Adp-ribose diphosphatase)(Adenosine diphosphoribose pyrophosphatase) (Adpr-ppase) (Adp-ribosephosphohydrolase) (Asppase) | Asppase | 20453 | 2 | 2 | 5 | 1 | 5 | 7 | 8 | 9 | 2.693 | 0.028 |
| D2N7R6 | 12322962 | Nodulation efficiency protein D (NfeD) | NfeD | 26001 | 1 | 1 | 1 | 1 | 4 | 2 | 2 | 4 | 2.631 | 0.013 |
| D2N4E3 | 12320668 | Phosphoglycerate mutase family protein | Phosphoglycerate mutase family protein | 22791 | 3 | 1 | 3 | 1 | 4 | 6 | 6 | 6 | 2.586 | 0.017 |
| D2N7R2 | 12322958 | PhoH family protein | PhoH family protein | 34913 | 4 | 2 | 7 | 7 | 11 | 12 | 13 | 17 | 2.551 | 0.02 |
| D2N5I8 | 12321074 | Ribonuclease R | Rnr | 90337 | 12 | 6 | 16 | 13 | 28 | 33 | 25 | 31 | 2.516 | 0.021 |
| D2N7Z5 | 12321745 | DNA-3-methyladenine glycosylase I | Tag | 21428 | 7 | 3 | 6 | 3 | 9 | 10 | 13 | 17 | 2.478 | 0.02 |
| D2N9Z1 | 12321385 | Zinc-binding lipoprotein AdcA | Zinc-binding lipoprotein AdcA | 59102 | 11 | 5 | 7 | 11 | 11 | 34 | 22 | 14 | 2.474 | 0.038 |
| D2N4S0 | 12320796 | Dihydroneopterin aldolase | FolB | 13691 | 3 | 1 | 1 | 3 | 4 | 4 | 9 | 3 | 2.410 | 0.036 |
| D2N363 | 12323015 | YycI protein | YycI protein | 29858 | 1 | 2 | 1 | 1 | 2 | 3 | 3 | 3 | 2.390 | 0.022 |
| D2NA39 | 12321433 | ABC transporter ATP-binding protein | ABC transporter ATP-binding protein | 25138 | 4 | 1 | 5 | 3 | 9 | 5 | 12 | 5 | 2.362 | 0.038 |
| D2N499 | 12320624 | NAD-dependent epimerase/dehydratase | NAD-dependent epimerase/dehydratase | 37815 | 26 | 18 | 14 | 15 | 66 | 36 | 45 | 28 | 2.359 | 0.021 |
| D2N6R1 | 12322607 | CDP-diacylglycerol--glycerol-3-phosphate 3-phosphatidyltransferase | PgsA | 21014 | 1 | 2 | 2 | 1 | 3 | 4 | 3 | 5 | 2.342 | 0.019 |
| D2N5Y1 | 12321217 | NAD kinase | NadK | 30769 | 3 | 2 | 2 | 4 | 7 | 7 | 8 | 6 | 2.337 | 0.019 |
| D2N804 | 12321754 | Probable GTP-binding protein EngB | EngB | 22685 | 2 | 1 | 2 | 2 | 3 | 5 | 5 | 4 | 2.180 | 0.017 |
| D2N421 | 12323323 | Putative N-acetylmannosamine-6-phosphate 2-epimerase | NanE | 24531 | 1 | 1 | 1 | 1 | 2 | 1 | 2 | 2 | 2.146 | 0.04 |
| D2N6C3 | 12322468 | Glutamate racemase | MurI | 29712 | 12 | 9 | 12 | 5 | 21 | 19 | 18 | 23 | 2.121 | 0.02 |
| D2N356 | 12323006 | DHH subfamily 1 protein | DHH subfamily 1 protein | 73766 | 10 | 6 | 11 | 5 | 19 | 15 | 12 | 21 | 2.110 | 0.021 |
| D2N8M1 | 12322007 | Methionine aminopeptidase | Map | 27444 | 5 | 9 | 10 | 14 | 19 | 21 | 25 | 16 | 2.100 | 0.021 |
| D2N5H2 | 12321057 | UPF0042 nucleotide-binding protein | UPF0042 nucleotide-binding protein | 34812 | 2 | 1 | 6 | 4 | 6 | 9 | 7 | 7 | 2.099 | 0.028 |
| D2N7R9 | 12322965 | Ribosomal RNA small subunit methyltransferase E | Ribosomal RNA small subunit methyltransferase E | 28411 | 2 | 2 | 2 | 2 | 3 | 5 | 5 | 5 | 2.098 | 0.011 |
| D2N870 | 12321820 | Ftsk/spoiiie family protein | Ftsk/spoiiie family protein | 144252 | 8 | 6 | 9 | 7 | 11 | 18 | 17 | 18 | 2.094 | 0.02 |
| D2N9Y2 | 12321376 | Nitrate reductase, alpha subunit | Nitrate reductase, alpha subunit | 139931 | 20 | 13 | 20 | 23 | 35 | 48 | 30 | 42 | 2.035 | 0.02 |
| D2N5V8 | 12321194 | 3-oxoacyl-[acyl-carrier-protein] synthase 3 | FabH | 33879 | 19 | 14 | 15 | 23 | 35 | 39 | 35 | 31 | 1.953 | 0.02 |
| D2NA13 | 12321407 | ABC transporter, ATP-binding/permease protein | ABC transporter, ATP-binding/permease protein | 65124 | 1 | 1 | 1 | 1 | 2 | 2 | 1 | 2 | 1.922 | 0.04 |
| D2N6V0 | 12322646 | Cardiolipin synthetase (Cardiolipin synthase) (CLsynthase) | Cardiolipin synthetase (Cardiolipin synthase) (CLsynthase) | 56451 | 4 | 3 | 1 | 2 | 6 | 4 | 5 | 5 | 1.917 | 0.028 |
| D2N6W2 | 12322658 | Catalase | Catalase | 58383 | 111 | 104 | 104 | 104 | 200 | 188 | 212 | 203 | 1.895 | 0.018 |
| D2N8Q5 | 12322041 | Aldehyde dehydrogenase | Aldehyde dehydrogenase | 51769 | 35 | 27 | 31 | 29 | 55 | 51 | 52 | 70 | 1.868 | 0.021 |
| D2N558 | 12320943 | Dihydroxyacetone kinase, DhaK subunit | DhaK | 34932 | 11 | 7 | 7 | 1 | 12 | 13 | 13 | 9 | 1.856 | 0.041 |
| D2N9V4 | 12321348 | TpgX protein | TpgX protein | 23317 | 34 | 32 | 33 | 31 | 74 | 43 | 58 | 65 | 1.844 | 0.021 |
| D2N860 | 12321810 | Formate--tetrahydrofolate ligase | Fhs | 59855 | 51 | 47 | 74 | 45 | 94 | 112 | 113 | 75 | 1.817 | 0.021 |
| D2N8R8 | 12322054 | Acyl-coenzyme A:6-aminopenicillanic acid acyl-transferase | Acyl-coenzyme A:6-aminopenicillanic acid acyl-transferase | 40391 | 4 | 1 | 4 | 2 | 6 | 5 | 4 | 6 | 1.796 | 0.037 |
| D2N5D0 | 12321015 | ABC transporter permease protein | ABC transporter permease protein | 56101 | 6 | 5 | 1 | 6 | 11 | 7 | 6 | 9 | 1.796 | 0.038 |
| D2N7M6 | 12322922 | Probable glycine dehydrogenase (decarboxylating) subunit 2 | GcvPB | 54793 | 8 | 9 | 6 | 12 | 21 | 15 | 18 | 10 | 1.795 | 0.043 |
| D2N694 | 12322439 | Glycerophosphoryl diester phosphodiesterase | Glycerophosphoryl diester phosphodiesterase | 34939 | 10 | 5 | 8 | 11 | 19 | 15 | 13 | 12 | 1.789 | 0.021 |
| D2N634 | 12322379 | Cytochrome aa3 quinol oxidase, subunit I | QoxB | 75242 | 23 | 19 | 26 | 27 | 37 | 32 | 45 | 57 | 1.778 | 0.021 |
| D2N4Q0 | 12320776 | 4-diphosphocytidyl-2-C-methyl-D-erythritol kinase | IspE | 31440 | 2 | 3 | 3 | 2 | 5 | 6 | 5 | 4 | 1.776 | 0.019 |
| D2N727 | 12322723 | Diaminopimelate decarboxylase | LysA | 47018 | 10 | 9 | 9 | 5 | 14 | 15 | 13 | 16 | 1.769 | 0.02 |
| D2N6R4 | 12322610 | Ribonuclease Y | Rny | 58512 | 21 | 16 | 15 | 13 | 26 | 26 | 28 | 32 | 1.737 | 0.02 |
| D2N9L2 | 12321256 | Molybdate ABC transporter, periplasmic molybdate-binding protein | ModA | 29072 | 13 | 26 | 17 | 32 | 35 | 41 | 43 | 32 | 1.710 | 0.029 |
| D2N5F5 | 12321040 | DegV family protein | DegV family protein | 32048 | 6 | 5 | 5 | 5 | 8 | 9 | 11 | 8 | 1.689 | 0.017 |
| D2N520 | 12320905 | Iron-binding protein | Iron-binding protein | 33286 | 3 | 3 | 4 | 6 | 9 | 6 | 7 | 6 | 1.663 | 0.037 |
| D2N9C8 | 12322279 | Alcohol dehydrogenase, zinc-binding domain protein | Alcohol dehydrogenase, zinc-binding domain protein | 37576 | 14 | 14 | 12 | 13 | 22 | 21 | 23 | 21 | 1.642 | 0.019 |
| D2N359 | 12323009 | Adenylosuccinate synthetase | PurA | 47551 | 5 | 11 | 9 | 4 | 10 | 15 | 12 | 12 | 1.640 | 0.042 |
| D2N6G7 | 12322513 | Isoleucine--tRNA ligase | IleS | 104941 | 78 | 65 | 72 | 96 | 107 | 119 | 120 | 158 | 1.621 | 0.021 |
| D2N9V2 | 12321346 | TagF domain protein | TagF domain protein | 65269 | 6 | 3 | 6 | 5 | 7 | 10 | 8 | 10 | 1.609 | 0.019 |
| D2N6Y5 | 12322681 | Transcription antiterminator | Transcription antiterminator | 32851 | 1 | 1 | 1 | 2 | 2 | 3 | 2 | 2 | 1.603 | 0.04 |
| D2N7P9 | 12322945 | ATP-dependent RNA helicase | ATP-dependent RNA helicase | 51107 | 14 | 10 | 15 | 18 | 26 | 23 | 20 | 22 | 1.600 | 0.021 |
| D2N4Q1 | 12320777 | Pur operon repressor | PurR | 28928 | 15 | 18 | 16 | 24 | 26 | 28 | 27 | 36 | 1.583 | 0.021 |
| D2N5F8 | 12321043 | Ribosomal subunit interface protein | Ribosomal subunit interface protein | 22213 | 17 | 17 | 27 | 20 | 28 | 30 | 34 | 36 | 1.573 | 0.02 |
| D2N662 | 12322407 | Potassium uptake protein TrkA | Potassium uptake protein TrkA | 24282 | 7 | 6 | 6 | 5 | 8 | 9 | 9 | 13 | 1.572 | 0.019 |
| D2N9C5 | 12322276 | Conserved domain protein | Conserved domain protein | 9223 | 16 | 13 | 13 | 14 | 24 | 19 | 21 | 22 | 1.565 | 0.02 |
| D2N7J7 | 12322893 | Glucose-6-phosphate 1-dehydrogenase | Zwf | 56965 | 41 | 32 | 53 | 40 | 53 | 64 | 68 | 75 | 1.558 | 0.029 |
| D2N3I0 | 12323132 | Acetoin(Diacetyl) reductase (Acetoin dehydrogenase) (AR)(Meso-2,3-butanediol dehydrogenase) | Acetoin(Diacetyl) reductase (Acetoin dehydrogenase) | 27215 | 31 | 29 | 33 | 30 | 45 | 37 | 48 | 59 | 1.551 | 0.021 |
| D2N780 | 12322776 | Asparagine--tRNA ligase | AsnS | 49157 | 53 | 37 | 52 | 48 | 70 | 65 | 76 | 79 | 1.535 | 0.021 |
| D2N8B2 | 12321862 | 2,5-diketo-D-gluconic acid reductase A | 2,5-diketo-D-gluconic acid reductase A | 31525 | 26 | 19 | 21 | 9 | 27 | 27 | 32 | 29 | 1.524 | 0.02 |
| D2N5Q3 | 12321139 | YumB | YumB | 44118 | 88 | 57 | 72 | 81 | 112 | 100 | 112 | 130 | 1.524 | 0.02 |
| D2N6S5 | 12322621 | DNA mismatch repair protein MutL | MutL | 76840 | 12 | 11 | 10 | 10 | 20 | 12 | 16 | 17 | 1.519 | 0.028 |
| D2N6M8 | 12322574 | DNA topoisomerase 1 | TopA | 79283 | 17 | 14 | 18 | 26 | 31 | 29 | 29 | 23 | 1.516 | 0.042 |
| D2N523 | 12320908 | Hydrolase, alpha/beta hydrolase fold family | Hydrolase, alpha/beta hydrolase fold family | 30955 | 10 | 10 | 13 | 10 | 17 | 16 | 16 | 15 | 1.513 | 0.017 |
| D2N4S2 | 12320798 | Lysine--tRNA ligase | LysS | 56719 | 87 | 73 | 93 | 93 | 95 | 123 | 162 | 141 | 1.507 | 0.02 |
| **Unknown proteins** | | | | | | | | | | | | | | |
| D2N9S7 | 12321321 | Uncharacterized protein | Uncharacterized protein | 24866 | 1 | 2 | 1 | 1 | 12 | 4 | 5 | 22 | 9.978 | 0.018 |
| D2N965 | 12322204 | Uncharacterized protein | Uncharacterized protein | 13339 | 1 | 1 | 1 | 1 | 9 | 6 | 5 | 6 | 8.447 | 0.013 |
| D2N3Y5 | 12323287 | Uncharacterized protein | Uncharacterized protein | 57881 | 1 | 1 | 1 | 18 | 27 | 36 | 19 | 32 | 5.510 | 0.018 |
| D2N899 | 12321849 | Uncharacterized protein | Uncharacterized protein | 57612 | 5 | 5 | 7 | 6 | 32 | 18 | 26 | 22 | 4.167 | 0.02 |
| D2N9W5 | 12321359 | Uncharacterized protein | Uncharacterized protein | 15066 | 1 | 1 | 1 | 1 | 3 | 3 | 2 | 4 | 3.982 | 0.013 |
| D2N4F3 | 12320678 | Uncharacterized protein | Uncharacterized protein | 25804 | 1 | 1 | 2 | 1 | 6 | 2 | 5 | 4 | 3.922 | 0.026 |
| D2N692 | 12322437 | Uncharacterized protein | Uncharacterized protein | 39639 | 1 | 1 | 1 | 1 | 2 | 1 | 3 | 4 | 3.245 | 0.047 |
| D2N5Y6 | 12321222 | Uncharacterized protein | Uncharacterized protein | 40154 | 1 | 1 | 3 | 3 | 4 | 6 | 6 | 7 | 2.689 | 0.019 |
| D2N5H8 | 12321064 | Uncharacterized protein | Uncharacterized protein | 24056 | 3 | 2 | 2 | 4 | 8 | 7 | 9 | 7 | 2.655 | 0.019 |
| D2N8E4 | 12321894 | Uncharacterized protein | Uncharacterized protein | 35950 | 4 | 5 | 2 | 6 | 7 | 13 | 18 | 7 | 2.598 | 0.02 |
| D2N678 | 12322423 | Uncharacterized protein | Uncharacterized protein | 15591 | 1 | 1 | 4 | 3 | 5 | 5 | 7 | 6 | 2.579 | 0.019 |
| D2N6V3 | 12322649 | Uncharacterized protein | Uncharacterized protein | 32911 | 11 | 10 | 15 | 11 | 27 | 17 | 22 | 34 | 2.150 | 0.02 |
| D2N9T5 | 12321329 | Uncharacterized protein | Uncharacterized protein | 24195 | 1 | 1 | 2 | 2 | 2 | 3 | 4 | 4 | 2.075 | 0.036 |
| D2N8T9 | 12322075 | Uncharacterized protein | Uncharacterized protein | 20617 | 2 | 1 | 1 | 3 | 5 | 3 | 5 | 3 | 2.027 | 0.036 |
| D2N8R7 | 12322053 | Uncharacterized protein | Uncharacterized protein | 6565 | 6 | 5 | 8 | 6 | 17 | 9 | 11 | 11 | 1.895 | 0.019 |
| D2N8B3 | 12321863 | Uncharacterized protein | Uncharacterized protein | 35045 | 29 | 27 | 22 | 40 | 51 | 43 | 41 | 61 | 1.648 | 0.021 |
| D2N6R0 | 12322606 | Uncharacterized protein | Uncharacterized protein | 15113 | 2 | 1 | 4 | 4 | 5 | 5 | 5 | 4 | 1.639 | 0.034 |
| D2N9C6 | 12322277 | Uncharacterized protein | Uncharacterized protein | 20758 | 55 | 35 | 64 | 64 | 103 | 83 | 84 | 75 | 1.582 | 0.02 |
| D2N971 | 12322210 | Uncharacterized protein | Uncharacterized protein | 52964 | 14 | 8 | 13 | 16 | 19 | 18 | 18 | 24 | 1.555 | 0.02 |
| D2N5K3 | 12321089 | Uncharacterized protein | Uncharacterized protein | 11193 | 4 | 6 | 4 | 5 | 9 | 8 | 9 | 6 | 1.667 | 0.027 |

**upplementary Tables**
