# Supplementary figures and images for: Distinct virulent network between healthcare- and community-associated Staphylococcus aureus based on proteomic analysis
Source: Clin Proteomics. 2018 Jan 8;15:2. doi: 10.1186/s12014-017-9178-5 (PMC5757299; doi:10.1186/s12014-017-9178-5)

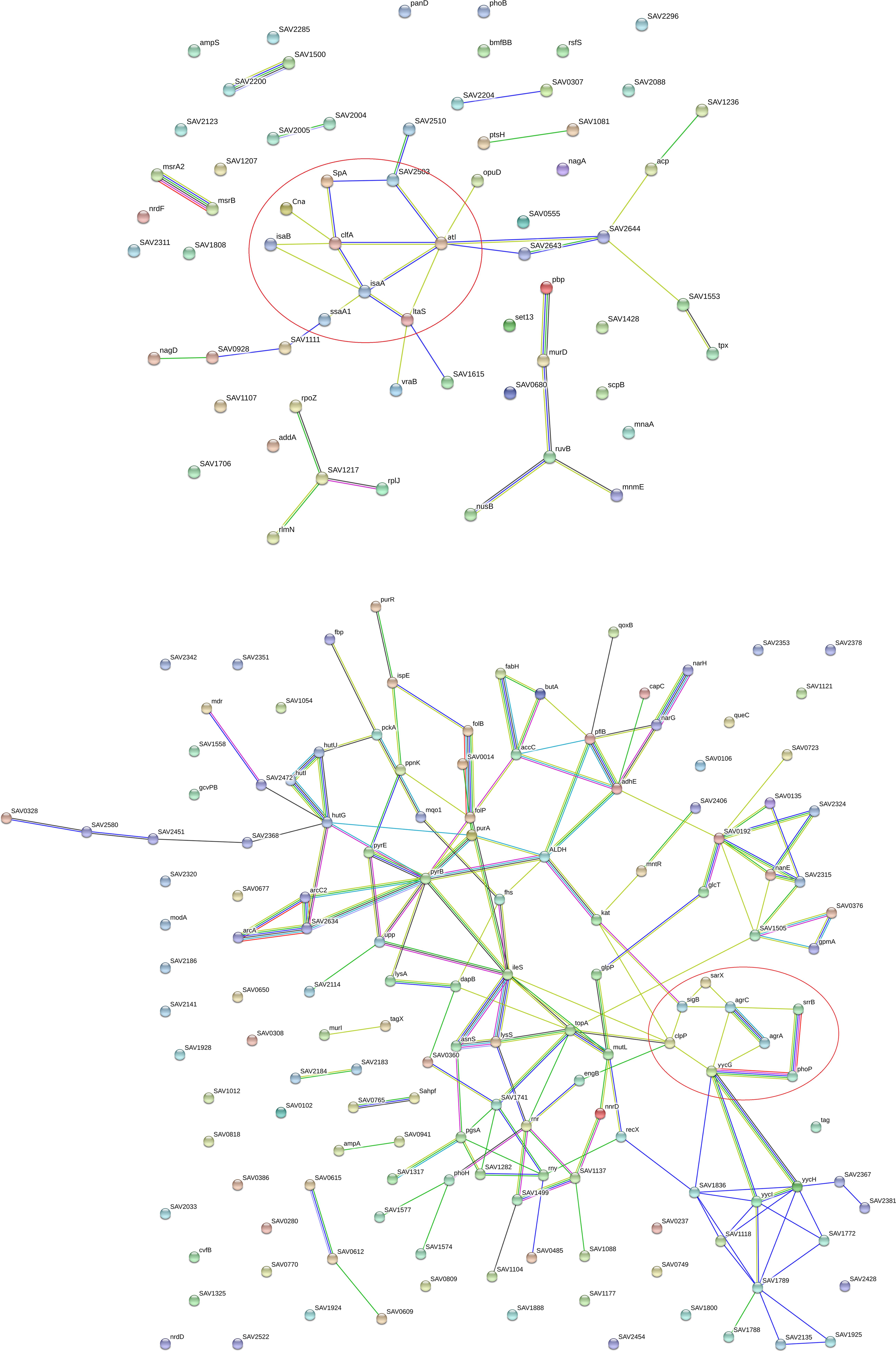

Supplement: Supplementary file 2 — Additional file 2. Potential interactions based on 64 up-regulated genes in the HA-SA ST239 group and 145 up-regulated genes in the CA-SA ST398 group using the STRING (version 10.0) database. [file 12014_2017_9178_MOESM2_ESM.jpg]
